# Design and Synthesis of Thiazolo[5,4-*f*]quinazolines as DYRK1A Inhibitors, Part I

**DOI:** 10.3390/molecules191015546

**Published:** 2014-09-29

**Authors:** Alicia Foucourt, Damien Hédou, Carole Dubouilh-Benard, Laurent Désiré, Anne-Sophie Casagrande, Bertrand Leblond, Nadège Loäec, Laurent Meijer, Thierry Besson

**Affiliations:** 1 Normandie Univ, Laboratoire C.O.B.R.A., UMR 6014 and FR 3038; Univ Rouen; INSA de Rouen; CNRS, Bâtiment I.R.C.O.F. rue Tesnière, Mont-Saint-Aignan F-76821, France; E-Mails: foucourtalicia@aol.com (A.F.); damien.hedou@etu.univ-rouen.fr (D.H.); carole.dubouilh@univ-rouen.fr (C.D.-B.); 2 Diaxonhit, 65 boulevard Masséna, Paris F-75013, France; E-Mails: laurent.desire@diaxonhit.com (L.D.); anne-sophie.casagrande@diaxonhit.com (A.-S.C.); bertrandleblond@hotmail.com (B.L.); 3 Protein Phosphorylation & Human Disease group, CNRS, Station Biologique, Roscoff F-29680, France; E-Mail: loaec@sb-roscoff.fr; 4 ManRos Therapeutics, Centre de Perharidy, Roscoff F-29680, France; E-Mail: meijer@manros-therapeutics.com

**Keywords:** thiazolo[5,4-*f*]quinazolines, kinases inhibitors, DYRK1A, GSK3α/β, microwave-assisted chemistry, Dimroth rearrangement, appel salt

## Abstract

The convenient synthesis of a library of novel 6,6,5-tricyclic thiazolo[5,4-*f*] quinazolines (forty molecules) was achieved mainly under microwave irradiation. Dimroth rearrangement and 4,5-dichloro-1,2,3,-dithiazolium chloride (Appel salt) chemistry were associated for the synthesis of a novel 6-aminobenzo[*d*]thiazole-2,7-dicarbonitrile (**16**) a versatile molecular platform for the synthesis of various bioactive derivatives. Kinase inhibition of the final compounds was evaluated on a panel of four Ser/Thr kinases (DYRK1A, CDK5, CK1 and GSK3) chosen for their strong implications in various regulation processes, especially Alzheimer’s disease (AD). In view of the results of this preliminary screening, thiazolo[5,4-*f*]quinazoline scaffolds constitutes a promising source of inspiration for the synthesis of novel bioactive molecules. Among the compounds of this novel chemolibrary, **7i**, **8i** and **9i** inhibited DYRK1A with IC_50_ values ranging in the double-digit nanomolar range (40, 47 and 50 nM, respectively).

## 1. Introduction

Kinases are one of the largest enzyme families of the genome. More than 500 kinases play an important role in the regulation of most cellular processes. These enzymes are involved in all major diseases, including cancer, neurodegenerative disorders and cardiovascular diseases [1,2,3]. Our research groups are mainly invested in the synthesis of C,N,S- or C,N,O-containing heterocyclic precursors of bioactive molecules able to modulate the activity of kinases in signal transduction [4,5,6,7,8].

In the course of our work based on microwave-assisted chemistry, we described ten years ago the multistep synthesis of the 8*H*-thiazolo[5,4-*f*]quinazolin-9-ones (**A**) [9,10]. Brief studies of their structure-activity relationships as dual CDK1/GSK-3 kinases inhibitors were described [7]. At that time, the inhibitory potency of the final products was evaluated and some products showed a micromolar range affinity against DYRK1A [11]. More recently, the synthesis and the kinase inhibitory potency of various benzo-, pyrido- and pyrazinothieno[3,2-*d*]pyrimidines derivatives (**B**), have been published. Kinase inhibition of the compounds was evaluated on Ser/Thr kinases (CDK5, GSK3, DYRK1A, CLK1 and CK1) selected for their strong implications in various human pathologies, especially in AD [3].

**Scheme 1:**
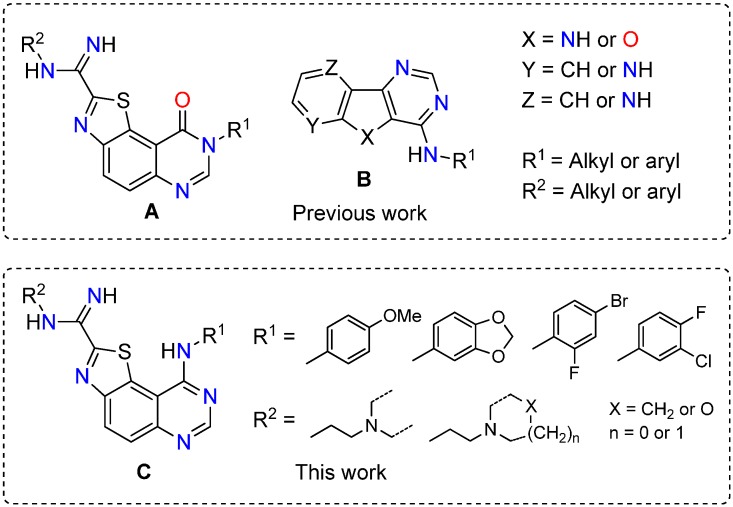
Structures of previous molecules which inspired the current work.

The overall pharmaceutical interest of all these compounds encouraged us to conceive new series of thiazolo[5,4-*f*]quinazolines substituted in position 4 of the pyrimidine ring by an aromatic amine and by carboximidamide groups in position 2 of the thiazole moiety (see general formula **C** in Scheme 1).

These compounds were conceived as 6,6,5-tricyclic homologues of the basic 4-aminoquinazoline pharmacophore which is present in approximately 80% of ATP-competitive kinase inhibitors that have received approval for the treatment of cancer [11]. The aromatic amine groups linked to the main thiazoloquinazoline structure were selected because of their frequent presence in drugs or drug candidates [11]. On the other side of the target molecules, the aliphatic chains of the carboximidamide groups were also chosen because of their frequent presence in many drugs.

This paper describes the development of a simple and reliable method that allows the preparation of a library of new thiazolo[5,4-*f*]quinazolines for which interesting kinase inhibitory activities were observed. The main part of the chemistry described in this paper was achieved under microwave irradiation as a continuation of our global strategy which consists to design adapted reactants and techniques offering operational, economic, and environmental benefits over conventional methods [12].

## 2. Results and Discussion

### 2.1. Synthesis

**Scheme 2:**
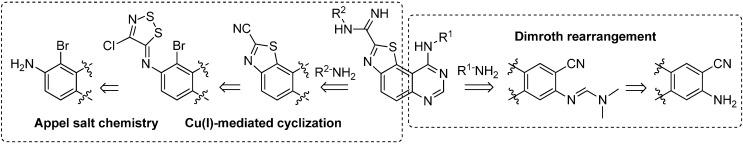
General retrosynthetic pathways envisioned for this work.

The target molecules we studied were thiazolo[5,4-*f*]quinazolines (**C**) substituted in position 4 of the pyrimidine ring (which corresponds to position 9 of the tricyclic compound) by an aromatic amine. The retrosynthetic pathway depicted in Scheme 2 was directly inspired by our previous work on the synthesis of various thiazoloquinazoline isomers [13,14] and on general access to pyrimidine-condensed heterocyclic compounds [15]. It suggested introducing the thiazole ring via a copper(I)-mediated cyclization of *ortho*-brominated *N*-arylimino-1,2,3-dithiazoles intermediates. The latter would be isolated after condensation of 4,5-dichloro-1,2,3-dithiazolium chloride (Appel salt) [16] with a key *N*^2^-protected brominated aminoanthranilonitrile. The synthesis of the final pyrimidinic structures was envisioned via a microwave-assisted thermal-sensitive Dimroth rearrangement. [17] A nucleophilic attack of intermediate amidines by various aromatic amines would give the expected tricyclic compounds. This fast and convenient procedure was recently explored for the design of novel bioactive 4-anilinoquinazolines [4,5,17].

The synthetic route described in Scheme 2 was applied to the preparation of some of our target molecules. It started from 2-amino-5-nitroanthranilonitrile (**1**) which was treated for 15 min at 105 °C with *N,N*-dimethylformamide dimethyl acetal (DMFDMA) under microwave irradiation. The resulting *N,N*-dimethylformamidine **2** was heated with the appropriate aromatic amine in the presence of acetic acid at 118 °C for 20 min. Compounds **3a**–**d** were then obtained in two steps in a good average yield of 85%.

In order to isolate the desired aryliminodithiazoles, 4-substituted-6-nitroquinazolinones **3a**–**c** were first reduced into 6-amino derivatives **4a**–**c** by transfer hydrogenation using ammonium formate in refluxing ethanol. Expectedly, in these conditions, the palladium-catalyzed reduction of the nitro group of intermediate **3d** also provoked the protodehalogenation of the chloride atom of the 3-chloro-4-fluoroaniline moiety. This difficulty was circumvented when the reduction of **3d** was achieved with iron and acetic acid in refluxing ethanol to give **4d** in excellent yield (99%).

**Scheme 3:**
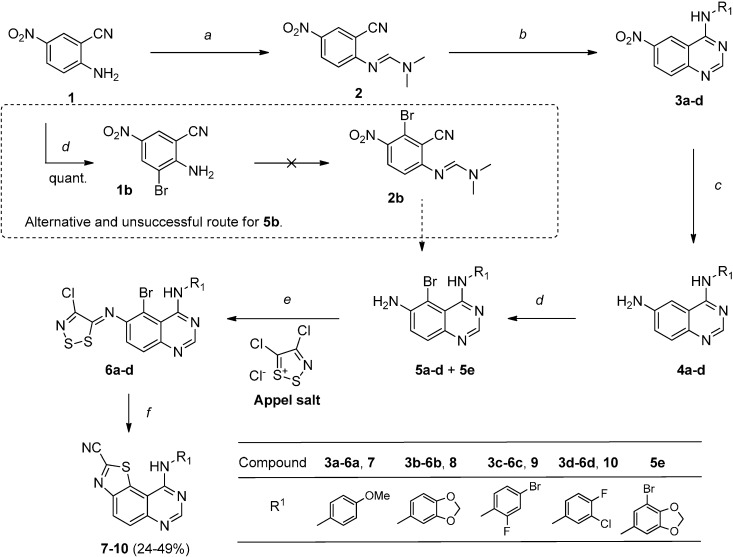
Synthetic routes experimented for the access to the target compounds (series **7**–**10**).

Compounds **4a**–**d** were treated with bromine in acetic acid to yield the *ortho* brominated imines **5a**–**d**. In the case of **4b**, a polybrominated by-product **5e** was obtained along with **5b** whatever the conditions tested. Unfortunately both derivatives **5b** and **5e** were inseparable under convenient conditions. Another route consisting in preliminary bromination of the starting 5-nitroanthranilonitrile (**1**) was experimented. Unfortunately, whatever the bromination method, only 3-bromo-5-nitroanthranilonitrile (**1b**) was detected (see alternative route in Scheme 3).

*Reagents and conditions*: (a) DMFDMA, DMF, 70 °C (μw), 2 min, 94%; (b) aniline (1.5 eq), AcOH, 118 °C (μw), 2 min, 99% (**3a**)/45 min, 95% (**3b**)/30 min, 70% (**3c**)/10 min, 77% (**3d**); (c) HCO_2_NH_4_, Pd.C, EtOH, 78 °C (μw), 30 min, 99% (**4a**)/, 99% (**4b**)/93% (**4c**) or Fe, AcOH, EtOH, 78 °C (μw), 5 h, 99% (**4d**); (d) Br_2_, AcOH, CH_2_Cl_2_, r.t., 3.5 h; quant.; (e) Appel salt, Py. (2 eq), CH_2_Cl_2_, r.t., 4 h, 39% (**6a**)/30% (**6c**)/39% (**6d**); (f) CuI, Py., 130 °C (μw), 20 min, 24% (**7**)/49% (**8**)/30% (**9**)/64% (**10**).

Brominated intermediates **5a**, **5c** and **5d** were condensed with 4,5-dichloro-1,2,3-dithiazolium chloride (Appel salt) in dichloromethane at room temperature and subsequent addition of pyridine led to the desired *ortho*-halogenated *N*-aryliminodithiazoles **6a**, **6c** and **6d**. The latter were converted into thiazolo[5,4-*f*]quinazoline-2-carbonitriles **7**, **9** and **10** in copper(I)-catalyzed conditions [14,16].

Despite its effectiveness, this synthesis presents some limitations. Each modification of the substituent in *N*^3^ of the pyrimidine ring (e.g., **3a**–**d**) generates three intermediates (e.g., **4a**–**d**, **5a**, **c** and **d** and **6a**–**d** in this study) for which synthetic and biological significance are not really established yet. The second drawback of this synthetic route lies in the reduction and bromination steps both of which require being adapted to the aromatic substituent of the intracyclic *N*^3^-nitrogen atom. As an example, compound **5b** was never isolated in analytically pure form and the synthesis of **8** via this intermediate was judged infeasible.

**Scheme 4:**
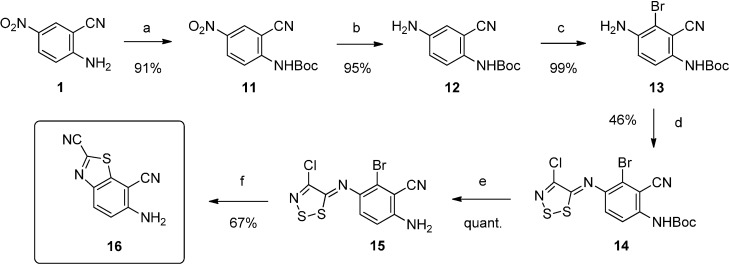
Multistep synthesis of polyfunctionalized benzothiazole **16**.

In order to avoid the limitations inherent in the first synthetic route and acquire an efficient and versatile route to various 9-anilinothiazolo[5,4-*f*]quinazolines, a rational multistep synthesis of a novel polyfunctionalized benzothiazole (see **16** in Scheme 4) was accomplished. This molecular system was designed as an efficient precursor of various target molecules.

*Reagents and conditions*: (a) Boc_2_O, DMAP, Et_3_N, CH_2_Cl_2_, r.t., 4 h; (b), HCO_2_NH_4_, Pd.C, EtOH, 78 °C (μw), 30 min; (c) Br_2_, AcOH, CH_2_Cl_2_, r.t., 2.5 h; (d) Appel salt, Py. (2 eq), CH_2_Cl_2_, r.t., 4 h; (e) AcOH, 118 °C (μw), 2 h; (f) CuI, Py., 130 °C (μw), 20 min.

Protection of 2-amino-5-nitrobenzonitrile (**1**) using di-*tert*-butyl dicarbonate was performed at room temperature in dichloromethane in the presence of triethylamine and 4-(dimethylamino)pyridine and provided *tert*-butyl (2-cyano-4-nitrophenyl)carbamate (**11**) in 91% yield. Reduction of the nitro group of **11** was carried out as previously shown by palladium-catalyzed transfer hydrogenation in refluxing ethanol under microwave irradiation for 30 min and gave *tert*-butyl 4-amino-2-cyanophenylcarbamate **12** in high yield. Treatment of intermediate **12** with a solution of bromine in dichloromethane in acetic acid at room temperature provided *tert*-butyl 4-amino-3-bromo-2-cyanophenylcarbamate **13** in quantitative yield. The latter was reacted with Appel salt (4,5-dichloro-1,2,3-dithiazolium chloride) in dichloromethane at room temperature to afford *tert*-butyl-3-bromo-4-(4-chloro-5*H*-1,2,3-dithiazol-5-ylideneamino)-2-cyanophenylcarbamate (**14**). *N*-Boc deprotection of **14** by acetic acid under microwave irradiation at 118 °C gave 6-amino-2-bromo-3-(4-chloro-5*H*-1,2,3-dithiazol-5-ylidene-amino)benzonitrile **15** in quantitative yield. Finally a copper-mediated cyclization of **15** was accomplished with CuI in pyridine under microwave irradiation at 130 °C for 20 min to give 6-aminobenzo[*d*]thiazole-2,7-dicarbonitrile **16** in good yield (67%).

**Scheme 5:**
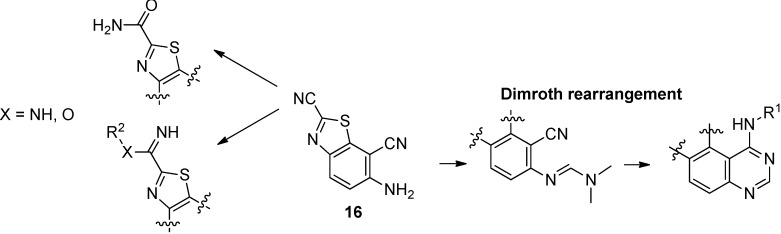
Possible transformations of benzothiazole **16** as a versatile molecular platform.

On a practical aspect, this synthetic sequence is easily upscalable and 10 g of 2-amino-5-nitro-benzonitrile (**1**) led to 2 g of **16** in an average 23% yield. This new compound **16** can be considered as a molecular platform that can be employed in new areas of investigation and prove its utility for the synthesis of innovative molecular systems with potent biological applications. Indeed, the versatile carbonitrile function in position 2 of the thiazole ring may allow the synthesis of various amidine, imidazoline and imidate derivatives. On the other side the 2-aminobenzonitrile moiety offers a large panel of possibilities for extension of the aromatic structure with a heterocyclic core such as a pyrimidine (Scheme 5).

The synthesis of the target molecules was carried on by treatment of **16** with DMFDMA under microwave irradiation at 70 °C to give (*E*)-*N*'-(2,7-dicyanobenzo[*d*]thiazol-6-yl)-*N,N*-dimethylformimidamide **(17**) in good yield (86%). Cyclization of formimidamide **17** into thiazolo[5,4-*f*]quinazoline-2-carbonitriles was accomplished via thermal Dimroth rearrangement using 1.5 eq of the appropriate aniline in acetic acid under microwave irradiation at 118 °C for short times and gave compounds **7**–**10** in good yields (71%–85%). This method constitutes a versatile route to various compounds, especially **8** which was obtained in excellent yield (98%).

In order to enhance the chemodiversity of the thiazolo[5,4-*f*]quinazolines studied, the reactivity of the aromatic carbonitriles **7**–**10** was tested against a panel of substituted amines (mainly alkylamines) inspired by our previous studies [4,5]. A new set of 27 novel carboximidamides **7a**–**g**, **8a**–**f**, **9a**–**g** and **10a**–**g** was prepared by stirring overnight at room temperature carbonitriles **7**–**10** with the appropriate amines (1.2 eq) in dry THF under argon. The chemical structures and yields obtained for the synthesis of the four prepared series (**7a**–**g**–**10a**–**g**) are shown in Table 1.

**Scheme 6:**
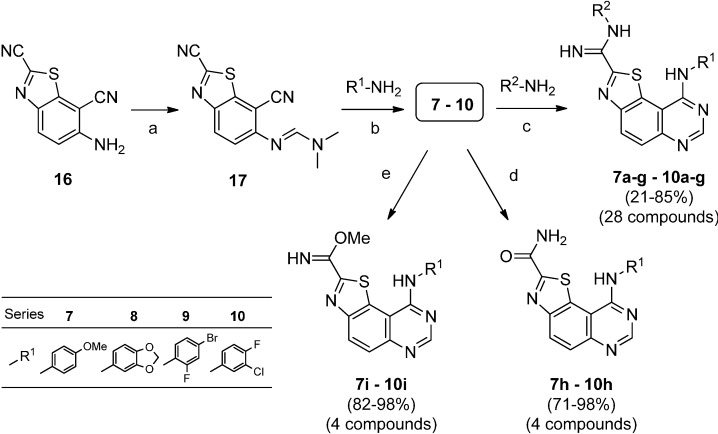
Synthesis of thiazolo[5,4-*f*]quinazoline-2-carbonitriles **7**–**10** and their derivatives via transformation of the carbonitrile functions in carboxamidines (**a**–**g**), amides (**h**) or imidates (**i**).

The key molecules **7**–**10** were also heated with sodium hydroxide (2.5 N in water) or with a solution of sodium methoxide in methanol to give respectively amides **7h**–**10h** and methyl imidates **7i**–**10i** in good to excellent yields (Scheme 6).

Note that microwave heating was mainly realized at atmospheric pressure in a controlled multimode cavity with a microwave power delivery system ranging from 0 to 1200 W. Concerning the technical aspect, the choice of a reactor able to work at atmospheric pressure was guided by our previous experience in microwave-assisted heterocyclic synthesis, especially in the chemistry of quinazolines [6,9]. Open vessel microwave experiments have some advantages, such as the possibility of easier scale-up and the possibility to use current laboratory glassware. Our choice was also guided by a recent work describing the tendency of pressure to accumulate when a product as DMFDMA was heated in pressurized vials, especially under microwaves [18]. In most cases of our study, an irradiation of 800 W was enough to efficiently reach the programmed temperature. This parameter was mainly monitored via a contactless-infrared pyrometer which was calibrated in control experiments with a fiber-optic contact thermometer.

*Reagents and conditions*: (a) DMFDMA, DMF, 70 °C (μw), 2 min, 86%; (b) aniline (1.5 eq), AcOH, 118 °C (μw), 2 min, 99% (**7**)/45 min, 95% (**8**)/30 min, 70% (**9**)/10 min, 77% (**10**); (c) amines, THF, r.t., 12 h, for yields see Table 1; (d) NaOH_aq_ (2.5 N), butanol, 117 °C (μw), 30 min, 98% (**7h**)/91% (**8h**)/71% (**9h**)/98% (**10h**); (e) NaOMe (0.5 M in MeOH), MeOH, 65 °C (μw), 30 min, 82% (**7i**)/92% (**8i**)/94% (**9i**)/98% (**10i**).

**Table 1:** Chemical structures and yields obtained for the synthesis of the four series (**7a**–**g**–**10a**–**g**).

R_1_	R_2_	Compound	Yield ^a^ (%)	R_1_	R_2_	Compound	Yield ^a^ (%)
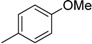	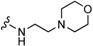	**7a**	41	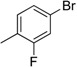	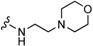	**9a**	85
	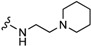	**7b**	43		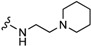	**9b**	72
	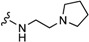	**7c**	47		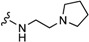	**9c**	68
	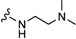	**7d**	53		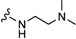	**9d**	64
	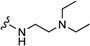	**7e**	50		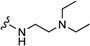	**9e**	86
	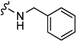	**7f**	28		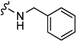	**9f**	68
	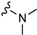	**7g**	67		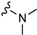	**9g**	40
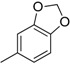	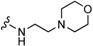	**8a**	41	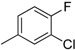	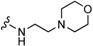	**10a**	71
	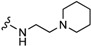	**8b**	34		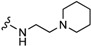	**10b**	82
	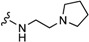	**8c**	48		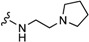	**10c**	69
	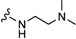	**8d**	30		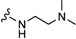	**10d**	50
	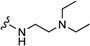	**8e**	66		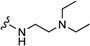	**10e**	50
	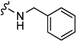	**8f**	21		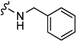	**10f**	69
	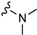	**8g**	-^b^		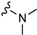	**10g**	43

^a^ Isolated yield; ^b^ Not prepared.

### 2.2. Biological Studies

Compounds of series **7** (**7**, **7a**–**i**), series **8** (**8**, **8a**–**i**), series **9** (**9**, **9a**–**i**) and series **10** (**10**, **10a**–**i**) were tested on four different *in vitro* kinase assays (CDK5/p25 (cyclin-dependent kinase), CK1δ/ε (casein kinase 1), GSK3α/β(Glycogen Synthase Kinase 3) and DYRK1A (dual-specificity, tyrosine phosphorylation regulated kinase) to evaluate their inhibition potency [19,20,21,22,23]. These four kinases are all involved in Alzheimer’s disease (AD), a multi-kinase inhibitor able to target two or three of them could be quite desirable. This is linked to the fact that it is still not known whether any of these four kinases plays a more prominent role in Alzheimer’s disease than the others and, consequently, which one should therefore preferably be targeted. In pathological situations such kinases are overexpressed and-activated, this fact justify the interest of multi-target-directed ligands (MTDLs) while complete inhibition is likely to be detrimental.

All compounds were first tested at a final concentration of 10 µM. Compounds showing less than 50% inhibition were considered as inactive (IC_50_ > 10 µM). Compounds displaying more than 50% inhibition at 10 µM were next tested over a wide range of concentrations (usually from 0.01 to 10 µM), and IC_50_ values were determined from the dose-response curves (Sigma-Plot). Harmine is a β-carboline alkaloid known to be a potent inhibitor of DYRK1A [24]. Leucettine L41 is also a potent DYRK1A inhibitor derived from a marine natural product, Leucettamine B [25,26]. They were tested as positive controls and their IC_50_ values were compared with those obtained for the compounds under study.

Results given in Table 2 demonstrate that none of the thiazolo[5,4-*f*]quinazoline derivatives exhibited any inhibitory activity against CDK5/p25 and CK1. Precursors **7**, **8**, **9** and **10**, were completely inactive against all four tested kinases (IC_50_ > 10 μM).

On a general aspect, compounds **a**–**f** of series **7**, **9** and **10** were also judged inactive against DYRK1A, except for some compounds (**8b**–**8e**) of series **8** for which micromolar IC_50_ values were observed. Note that among the four families of tested molecules, all compounds of **a**–**f** series were found relatively active (mainly in the micromolar range) against GSK3α/β. All these compounds were substituted with bulky aminoethyl groups on the carboximidamide function of the final structures. The large size of these groups appeared to influence the reactivity of the products against the four kinases tested, conferring them a relative selectivity for GSK3α/β (1.10 μM < IC_50_ < 7.00 μM).

**Table 2:** Kinase inhibitory activity ^a,b,c^ of the four thiazolo[5,4-*f*]quinazoline series (**7a**–**i**–**10a**–**i**).

Compound	DYRK1A	CK1	CDK5	GSK3	Compound	DYRK1A	CK1	CDK5	GSK3
**7**	>10	>10	>10	≥10	**9**	>10	>10	>10	>10
**7a**	>10	>10	>10	1.10	**9a**	>10	>10	>10	1.8
**7b**	>10	>10	>10	2.50	**9b**	>10	>10	>10	**0.53**
**7c**	>10	>10	>10	2.00	**9c**	>10	>10	>10	2.20
**7d**	>10	>10	>10	>10	**9d**	>10	>10	>10	**0.95**
**7e**	4.00	>10	>10	1.30	**9e**	>10	>10	>10	2.10
**7f**	8.00	>10	>10	2.00	**9f**	>10	>10	>10	1.80
**7g**	**0.70**	>10	>10	1.10	**9g**	**0.27**	>10	>10	**0.60**
**7h**	**0.50**	>10	>10	**0.30**	**9h**	**0.67**	>10	>10	**0.13**
**7i**	**0.040**	>10	>10	**0.20**	**9i**	**0.050**	>10	>10	**0.16**
**8**	>10	>10	>10	≥10	**10**	>10	>10	>10	>10
**8a**	2.20	>10	>10	**0.97**	**10a**	>10	>10	>10	3.50
**8b**	2.00	>10	>10	1.10	**10b**	>10	>10	>10	1.40
**8c**	1.10	>10	>10	**0.36**	**10c**	>10	>10	>10	2.50
**8d**	1.05	>10	>10	**0.25**	**10d**	>10	>10	>10	3.00
**8e**	6.50	>10	>10	**0.80**	**10e**	>10	>10	>10	7.00
**8f**	>10	>10	>10	2.00	**10f**	>10	>10	>10	>10
**8g**	-^d^	-	-	-	**10g**	6.50	>10	>10	7.20
**8h**	**0.80**	>10	>10	**0.77**	**10h**	1.60	>10	>10	**0.66**
**8i**	**0.047**	>10	>10	**0.66**	**10i**	**0.25**	>10	>10	**0.69**

^a^ IC_50_ values are reported in μM. The most significant results are presented in bold; ^b^ Kinases activities were assayed in triplicate. Typically, the standard deviation of single data points was below 10%; ^c^ Harmine (IC_50_ in μM): DYRK1A: 0.029; CK1: 1.50; CDK5 and GSK3α/β: > 10 [27]; Leucettine L41 (IC_50_ in μM): DYRK1A: 0.040; CK1: > 10; CDK5: > 10 and GSK3α/β: 0.040 [27]; ^d^ Not determined.

Among the compounds tested, the two most interesting series are **8** and **9** which showed submicromolar values against GSK3 α/β. From this point of view, series **8** is really promising with micromolar range activities against DYRK1A (6.5 μM < IC_50_ < 1.05 μM) and submicromolar IC_50_ values against GSK3α/β (0.25 μM < IC_50_ < 0.97 μM).

Undoubtedly, the most active molecules prepared in this study were series **g**–**i** of the four family of thiazolo[5,4-*f*]quinazolines (**7**–**10**). The latter showed spectacular submicromolar activities against DYRK1A (0.04 μM < IC_50_ < 0.70 μM) and GSK3α/β kinases (0.16 μM < IC_50_ < 0.77 μM) with a marked preference for the first one, respectively. The DYRK1A IC_50_ values obtained for **7i**, **8i** and **9i** are situated in the double-digit nanomolar range (40, 47 and 50 nM, respectively) demonstrating that small-sized groups linked to the thiazole ring were able to induce a dramatic enhancement of the inhibitory activity against DYRK1A.

Taking into account these preliminary results, defining a specific role of the aromatic substituents of the amine located at position 4 of the pyrimidine ring remains challenging. Nevertheless, the presence of substituting groups on the aromatic moiety seemed to have a positive effect on the inhibitory activity of the studied compounds. Without being able to establish a general rule, the presence of substituents in positions 2 and 4 of the aromatic chain (series **9**) has a rather beneficial effect compared to substituents in position 3 and 4 (series **8** and **10**).

The most active thiazoloquinazolines were less potent and specific against DYRK1A (Table 2) compared to harmine and leucettine L41 [25,26,27] but constitute a promising source of inspiration for the synthesis of novel bioactive molecules. Our results confirm that the thiazolo[5,4-*f*]quinazoline scaffold has a great potential in the development of potent inhibitors of DYRK1A and GSK3α/β kinases that are involved in many neurodegenerative diseases and cancers. Lead compounds **7i**, **8i** and **9i** presented in this paper allow us to consider further structure-activity relationships for the design of more efficient and selective inhibitors of these targeted kinases.

## 3. Experimental Section

### 3.1. General Information

All reactions were carried out under inert atmosphere of argon or nitrogen and monitored by thin-layer chromatography with silica gel 60 F254 pre-coated aluminum plates (0.25 mm). Visualization was performed with a UV light at 254 and 312 nm. Purifications were carried out on an Armen Instrument Spot 2 Flash System equipped with a dual UV-Vis spectrophotometer (200–600 nm), a fraction collector (192 tubes), a dual piston pump (1 to 250 mL/min, P_max_ = 50 bar/725 psi) allowing quaternary gradients and an additional inlet for air purge. Samples can be injected in liquid or solid mode. Purification was edited and monitored on an integrated panel PC with a touch screen controlled by Armen Glider Flash v3.1d software [28]. Biotage SNAP flash chromatography cartridges (KP-Sil, normal phase, 10 to 340 g) were used for the purification process. Melting points of solid compounds were measured on a WME Köfler hot-stage with a precision of +/−2 °C and are uncorrected. IR spectra were recorded on a PerkinElmer Spectrum 100 Series FT-IR spectrometer. Liquids and solids were applied on the Single Reflection Attenuated Total Reflectance (ATR) Accessories. Absorption bands are given in cm^−1^.

^1^H/^19^F/^13^C-NMR spectra were recorded on a Bruker DXP 300 spectrometer at 300, 282 and 75 MHz respectively. Abbreviations used for peak multiplicities are s: singlet, d: doublet, t: triplet, q: quadruplet and m: multiplet. Coupling constants *J* are in Hz and chemical shifts are given in ppm and calibrated with DMSO-*d*_6_ or CDCl_3_ (residual solvent signals). Mass spectra analysis was performed by the Mass Spectrometry Laboratory of the University of Rouen. Mass spectra (EI) were recorded with a Waters LCP 1er XR spectrometer.

Dichloromethane was distilled from CaH_2_ under argon. NBS was recrystallized in water. Other reagents and solvents were used as provided by commercial suppliers.

Appel salt was prepared according to literature procedure [16] by addition of chloroacetonitrile (1 eq) to a solution of sulfur dichloride (5 eq) in dichloromethane (50 mL). Adogen™ (3–4 drops) was then added and the reaction was placed in a bowl of cold water. The mixture was left for 18 h without stirring under CaCl_2_ tube protection: The dark olive green solid was removed from the wall of the flask, filtered off under a blanket of argon, washed abundantly with dichloromethane and dried under vacuum for 2–3 h (average yield: 85%): mp 172–174 °C (dec); IR (nujol) cm^−1^ 1707, 1358 s, 1280 s, 1253, 1083, 917, 828 s, and 605.

Microwave experiments were conducted at atmospheric pressure in a commercial microwave reactors especially designed for synthetic chemistry. Time indicated in the various protocols is the time measured when the mixtures reached the programmed temperature after a ramp period of 2 min. RotoSYNTH™ (Milestone S.r.l. Italy) is a multimode cavity with a microwave power delivery system ranging from 0 to 1200 W. Open vessel experiments were carried out in round bottom flask (from 25 mL to 4 L) fitted with a reflux condenser. The temperature was monitored via a contact-less infrared pyrometer (IRT) and fiber-optic contact thermometer (FO). Temperature, pressure and power profiles were edited and monitored through the EASY-Control software provided by the manufacturer. Compounds **7a**–**i**, **8a**–**i**, **9a**–**i** and **10a**–**i**, **16** and **17** were described in a previous patent application [29]; to help readers, physicochemical data of these compounds are added in the following experimental part.

### 3.2. Synthesis

#### 3.2.1. Synthesis of Formamidine **2** and Amines **3a**, **3c** and **3d**

*N*'-(2-Cyano-4-nitrophenyl)-*N*,*N*-dimethylformamidine (**2**), *N*-(4-methoxyphenyl)-6-nitro-quinazolin-4-amine (**3a**), *N*-(4-bromo-2-fluorophenyl)-6-nitroquinazolin-4-amine (**3c**) and *N*-(3-chloro-4-fluorophenyl)-6-nitroquinazolin-4-amine (**3d**) were prepared and characterized following the general procedure described in [6].

*N-(Benzo[d][1,3]dioxol-5-yl)-6-nitroquinazolin-4-amine* (**3b**). A mixture of *N'*-(2-cyano-4-nitrophenyl)-*N,N*-dimethylformamidine (**2**, 1.0 g, 4.58 mmol) and 3,4-(methylenedioxy)aniline (0.63 g, 4.58 mmol) in acetic acid (5 mL) was heated at 118 °C under microwaves (600 W). On completion (followed by TLC or GC-MS), the reaction was cooled to ambient temperature. The separated solid was filtered and washed with diethyl ether to obtain the expected compound **3b** (1.08 g, 76%) as a brown solid; mp > 260 °C. IR (cm^−1^) ν*_max_* 1581, 1512, 1490, 1321, 1245, 1222, 1188, 1031, 921, 896, 846, 807; ^1^H-NMR (DMSO-*d*_6_) δ 10.3 (s, 1H, NH), 9.57 (d, 1H, *J* = 2.1 Hz), 8.64 (s, 1H), 8.52 (dd, 1H, *J*_1_ = 2.1 Hz, *J*_2_ = 9.0 Hz), 7.88 (d, 1H, *J* = 9.0 Hz), 7.44 (s, 1H), 7.18 (m, 1H), 6.96 (d, 1H, *J* = 9.0 Hz), 5.75 (s, 2H); ^13^C-NMR (DMSO-*d*_6_) δ 158.6, 157.6, 152.9, 146.8, 144.2, 144.1, 132.3, 129.2, 126.2, 120.5, 116.1, 114.1, 107.6, 105.0, 101.2; HRMS calcd for C_15_H_11_N_4_O_4_ [M+H]^+^ 311.0780, found 311.0794.

#### 3.2.2. Synthesis of Diamines **4b**, **4c** and **4d**

General procedure: a stirred mixture of **3b**, **3c** and **3d** (1 mmol), ammonium formate (5 mmol) and a catalytic amount of 10% palladium charcoal in 20 mL of ethanol was heated at reflux (600 W) for 30 min. The catalyst was removed by filtration through Celite and washed with ethanol. The resulting filtrate was evaporated under reduced pressure to give **4b**, **4c** and **4d**.

*N^4^-(Benzo[d][1,3]dioxol-5-yl)quinazoline-4,6-diamine* (**4b**). Prepared from **36** (quant. yield); brown solid; mp = 112–114 °C. IR (KBr) ν*_max_*/cm^−1^ 3339, 2976, 1619, 1572, 1530, 1499, 1448, 1384, 1343, 1255, 1190, 1095, 1045, 932, 882, 823; ^1^H-NMR (DMSO-*d*_6_) δ 8.20 (s, 1H), 7.51 (dd, 2H, *J*_1_ = 2.0 Hz, *J*_2_ = 9.0 Hz), 7.32 (d, 1H, *J* = 2.0 Hz), 7.23–7.18 (m, 2H), 6.91 (d, 1H, *J* = 9.0 Hz), 6.00 (s, 2H); ^13^C-NMR (DMSO-*d*_6_) δ 163.7, 156.3, 149.9, 147.3, 146.9, 143.2, 142.1, 134.0, 128.4, 123.6, 116.6, 115.2, 107.8, 104.6, 101.1; HRMS calcd for C_15_H_13_N_4_O_2_ [M+H]^+^: 281.1039, found 281.1033.

*N^4^-(4-Bromo-2-fluorophenyl)quinazoline-4,6-diamine* (**4c**). Prepared from **3c** (yield: 93%); brown solid; mp = 216–218 °C. IR (cm^−1^) ν*_max_* 3245, 3067, 1923, 1614, 1561, 1533, 1493, 1424, 1390, 1335, 1237, 1206, 1070, 887, 846, 825; ^19^F-NMR (DMSO-*d*_6_) δ −115.6; ^1^H-NMR (DMSO-*d*_6_) δ 8.53 (s, 1H), 7.72 (d, 1H, *J* = 9.9 Hz), 7.62 (d, 1H, *J* = 8.7 Hz), 7.52 (m, 2H), 7.40–7.36 (m, 2H); ^13^C-NMR (DMSO-*d*_6_) δ 158.8, 158.4, 155.1, 149.6, 146.6, 131.2, 130.1, 128.0, 127.9, 126.0, 124.7, 124.5, 121.7, 120.0, 119.9, 119.8, 119.5, 115.1, 101.4; HRMS calcd for C_14_H_11_N_4_BrF [M+H]^+^: 334.0151, found 334.0158.

*N^4^-(3-Chloro-4-fluorophenyl)quinazoline-4,6-diamine* (**4d**). Prepared from **3d** (99% yield); brown solid; mp = 244–246 °C. IR (cm^−1^) ν*_max_* 2501, 1633, 1614, 1568, 1503, 1432, 1373, 1266, 1214, 888, 871, 794; ^19^F-NMR (DMSO-*d*_6_) δ −123.9; ^1^H-NMR (DMSO-*d*_6_) δ 9.48 (s, 1H, NH), 8.36 (s, 1H), 8.21 (dd, 1H, *J*_1_ = 2.4 Hz, *J*_2_ = 6.9 Hz), 7.85–7.80 (m, 1H), 7.55 (d, 1H, *J* = 9.3 Hz), 7.41 (t, 1 H, *J* = 9.3 Hz); 7.30–7.24 (m, 2H); ^13^C-NMR (DMSO-*d*_6_) δ 155.7, 154.5, 151.3, 149.5, 147.5, 142.6, 137.4, 137.3, 128.7, 123.8, 122.8, 121.8, 121.7, 118.8, 188.6, 116.7, 116.6, 116.3, 100.9; HRMS calcd for C_14_H_11_N_4_ClF [M+H]^+^: 289.0656, found 289.0663.

#### 3.2.3. Synthesis of Brominated Diamines **5a**, **5c** and **5d**

General procedure: a solution of bromine (2.9 mmol) in dichloromethane (3 mL) was added dropwise, under an argon atmosphere, to a solution of amines **4a**, **4c** and **4d** (2.9 mmol) in acetic acid (34 mL). After 3.5 h of stirring at room temperature, the solvent was removed *in vacuo*. Excess acetic acid was co-evaporated with heptane to afford the expected compounds **5a**, **5c** and **5d**.

*5-Bromo-N^4^-(4-methoxyphenyl)quinazoline-4,6-diamine* (**5a**). Prepared from **4a** (quant. Yield), beige solid; mp > 260 °C. IR (cm^−1^) ν*_max_* 3297, 3106, 3065, 2950, 2668, 1607, 1567, 1510, 1381, 1356, 1299, 1229, 1168, 1073, 1024, 820, 802; ^1^H-NMR (DMSO-*d*_6_) δ 10.54 (s, 1H, NH), 8.69 (s, 1H), 7.71–7.68 (m, 2H), 7.64 (d, 2H, *J* = 9.0 Hz), 7.03 (d, 2H, *J* = 9.0 Hz), 3.78 (s, 3H); ^13^C-NMR (DMSO-*d*_6_) δ 157.7, 157.1, 147.9, 145.9, 131.7, 129.3, 125.6, 125.2, 119.4, 114.1, 113.0, 96.3, 55.5; HRMS calcd for C_15_H_14_N_4_OBr [M+H]^+^: 345.0351, found 345.0367.

*5-Bromo-N^4^-(4-bromo-2-fluorophenyl)quinazoline-4,6-diamine* (**5c**). Prepared from **4c** (quant. Yield), yellow solid; mp > 260 °C. IR (cm^−1^) ν*_max_* 3369, 3334, 3256, 1619, 1565, 1514, 1477, 1432, 1410, 1390, 1327, 1262, 1235, 1179, 1165, 1100, 1063, 954, 928, 875, 857, 828, 815; ^19^F-NMR (DMSO-*d*_6_) δ −119.3; ^1^H-NMR (DMSO-*d*_6_) δ 8.46 (s, 1H), 8.01 (t, 1H, *J* = 9.0 Hz), 7.69–7.61 (m, 2H), 7.53 (d, 1H, *J* = 9.0 Hz), 7.47 (m, 1H); ^13^C-NMR (DMSO-*d*_6_) δ 156.3, 155.9, 152.9, 147.0, 137.3, 127.5, 126.8, 124.9, 123.5, 118.9, 118.6, 117.5, 113.6, 94.9; HRMS calcd for C_14_H_10_N_4_Br_2_F [M+H]^+^: 410.9256, found 410.9243.

*5-Bromo-N^4^-(3-chloro-4-fluorophenyl)quinazoline-4,6-diamine* (**5d**). Prepared from **4d** (quant. Yield), orange solid; mp > 260 °C. IR (cm^−1^) ν*_max_* 3464, 3319, 3286, 3185, 2736, 1705, 1626, 1566, 1519, 1495, 1425, 1384, 1360, 1329, 1302, 1267, 1214, 1159, 1136, 1054, 930, 872, 826; ^19^F-NMR (DMSO-*d*_6_) δ −118.8; ^1^H-NMR (DMSO-*d*_6_) δ 10.51 (s, 1H, NH), 8.75 (s, 1H), 8.04 (dd, 1H, *J*_1_ = 2.4 Hz, *J*_2_ = 6.9 Hz), 7.71–7.68 (m, 2H), 7.63 (d, 1H, *J* = 9.0 Hz), 7.55 (t, 1H, *J* = 9.0 Hz); ^13^C-NMR (DMSO-*d*_6_) δ 157.4, 156.8, 153.5, 148.1, 145.9, 133.8, 133.7, 132.5, 126.1, 125.6, 125.0, 124.9, 119.8, 119.5, 119.3, 117.2, 116.9, 113.3, 95.9; HRMS calcd for C_14_H_10_N_4_BrClF [M+H]^+^: 366.9761, found 366.9770.

#### 3.2.4. Synthesis of Imino-1,2,3-dithiazoles **6a**, **6c** and **6d**

General procedure: a suspension of the 5-bromoquinazoline **5a**, **5c** and **5d**. (10 mmol), 4,5-dichloro-1,2,3-dithiazolium chloride **12** (22 mmol) in dichloromethane (50 mL) was stirred at room temperature under an argon atmosphere. After 3 h of stirring at room temperature, pyridine (44 mmol) was added and the mixture was stirred again for 1 h at room temperature. Then the resulting solution was concentrated under reduced pressure. The obtained crude residue was purified by flash chromatography (DCM–EtOAc, 8:2) to afford the expected compounds **6a**, **6c** and **6d**.

*(Z)-5-Bromo-N^6^-(4-chloro-5H-1,2,3-dithiazol-5-ylidene)-N^4^-(4-methoxyphenyl)quinazoline-4,6-diamine* (**6a**). Prepared from **5a** (yield: 39%), brown solid; mp > 260 °C. IR (cm^−1^) ν*_max_* 3239, 1607, 1567, 1504, 1440, 1377, 1303, 1249, 1172, 1027, 830; ^1^H-NMR (DMSO-*d*_6_) δ 10.93 (s, 1H, NH), 8.96 (s, 1H), 7.95–7.85 (m, 2H), 7.66 (d, 2H, *J* = 9.0 Hz), 7.07 (d, 2H, *J* = 9.0 Hz), 3.79 (s, 3H); HRMS calcd for C_17_H_12_N_5_OS_2_BrCl (M + H^+^): 479.9355, found 479.9362.

*(Z)-5-Bromo-N^4^-(4-bromo-2-fluorophenyl)-N^6^-(4-chloro-5H-1,2,3-dithiazol-5-ylidene)quinazoline-4,6-diamine* (**6c**). Prepared from **5c** (yield: 30%), yellow solid; mp = 198–200 °C. IR (cm^−1^) ν*_max_* 3336, 3086, 1704, 1621, 1610, 1593, 1530, 1489, 1478, 1407, 1389, 1314, 1237, 1180, 1146, 1109, 945, 921, 859, 852, 835, 818, 810; ^19^F-NMR (DMSO-*d*_6_) δ −118.3; ^1^H-NMR (DMSO-*d*_6_) δ 9.78 (s, 1H, NH), 8.57 (s, 1H), 7.98 (m, 1H), 7.91 (d, 1H, *J* = 9.0 Hz), 7.76 (d, 1H, *J* = 9.0 Hz), 7.70 (m, 1H), 7.49 (m, 1H); HRMS calcd for C_16_H_8_N_5_S_2_Br_2_ClF [M+H]^+^: 545.8260, found 585.8273.

*(Z)-5-Bromo-N^4^-(3-chloro-4-fluorophenyl)-N^6^-(4-chloro-5H-1,2,3-dithiazol-5-ylidene)quinazoline-4,6-diamine* (**6d**). Prepared from **5d** (yield: 39%), orange solid; mp = 218–220 °C. IR (cm^−1^) ν*_max_* 3353, 2980, 1571, 1534, 1492, 1414, 1383, 1337, 1261, 1207, 1153, 1054, 960, 921, 869, 803; ^19^F-NMR (DMSO-*d*_6_) δ−118.1; ^1^H-NMR (DMSO-*d*_6_) δ 9.90 (s, 1H, NH), 8.62 (s, 1H), 8.08 (dd, 1H, *J*_1_ = 2.4 Hz, *J*_2_ = 6.9 Hz), 7.93 (d, 1H, *J* = 9.0 Hz), 7.77 (d, 1H, *J* = 9.0 Hz), 7.71–7.66 (m, 1H), 7.46 (t, 1H, *J* = 9.0 Hz); HRMS calcd for C_16_H_8_N_5_S_2_BrCl_2_F [M+H]^+^: 501.8765, found 501.8766.

#### 3.2.5. Synthesis of 6-Aminobenzo[*d*]thiazole-2,7-dicarbonitrile (**16**) and Its Dimethylformimidamide Derivative (**17**)

*tert-Butyl 2-cyano-4-nitrophenylcarbamate* (**11**). To a solution of 2-amino-5-nitrobenzonitrile (**1**) (10.0 g, 61.3 mmol) in dichloromethane (100 mL) were added triethylamine (8.50 mL, 61.3 mmol), di-*tert*-butyl dicarbonate (26.8 g, 123 mmol), and 4-(dimethylamino)pyridine (7.50 g, 61.3 mmol). The solution was stirred for 4 h at room temperature under an argon atmosphere. The solvent was removed *in vacuo* and the crude residue was purified by flash chromatography (DCM-petroleum ether, 8:2) to afford the expected compound **11** (14.6 g, 91% yield) as a white solid; mp = 134–136 °C. IR (cm^−1^) ν*_max_* 3412, 3072, 3012, 2982, 2935, 2229, 1735, 1617, 1582, 1543, 1508, 1473, 1455, 1420, 1372, 1350, 1320, 1303, 1257, 1234, 1176, 1143, 1052, 1028, 923, 915, 889, 853; .^1^H-NMR (DMSO-*d*_6_) δ 10.04 (s, 1H, NH), 8.63 (d, 1H, *J* = 2.7 Hz), 8.44–8.40 (dd, 1H, *J*_1_ = 2.7 Hz, *J*_2_ = 9.3 Hz), 7.85 (d, 1H, *J* = 9.3 Hz), 1.49 (s, 9H); ^13^C-NMR (DMSO-*d*_6_) δ 153.1, 146.6, 142.6, 129.3, 128.8, 123.4, 115.2, 105.3, 81.2, 27.3; HRMS calcd for C_12_H_12_N_3_O_4_ [M+H]^+^: 262.0828, found 262.0831.

*tert-Butyl 4-amino-2-cyanophenylcarbamate* (**12**). A stirred mixture of **11** (10.0 g, 37.9 mmol), ammonium formate (189.5 mmol) and palladium charcoal (10%) in 300 mL of ethanol was heated at reflux under microwaves (600 W) for 30 min. The catalyst was removed by filtration through Celite and washed with ethanol. The resulting filtrate was evaporated under reduced pressure. Then, the residue was dissolved in EtOAc, washed with water, dried over MgSO_4_ and concentrated under reduced pressure to give the reduced compound **12** (8.4 g, 95% yield) as a pale yellow solid, mp = 126–128 °C. IR (cm^−1^) ν*_max_* 3476, 3431, 3365, 3398, 2988, 2934, 2222, 1697, 1628, 1587, 1521, 1443, 1429, 1392, 1367, 1324, 1294, 1274, 1250, 1230, 1161, 1053, 1028, 947, 902, 872, 849, 824; ^1^H-NMR (DMSO-*d*_6_) δ 8.82 (s, 1H, NH), 7.02 (d, 1H, *J* = 8.1 Hz), 6.81 (s, 1H), 6.78 (d, 1H, *J* = 2.7 Hz), 5.44 (s, 2H, NH_2_), 1.43 (s, 9H); ^13^C-NMR (DMSO-*d*_6_) δ 153.8, 146.7, 128.8, 127.9, 118.7, 117.5, 115.9, 109.8, 79.0, 28.0; HRMS calcd for C_12_H_16_N_3_O_2_ [M+H]^+^: 234.1243, found 234.1240.

*tert-Butyl 4-amino-3-bromo-2-cyanophenylcarbamate* (**13**). A solution of bromine (25.1 mmol) in dichloromethane (1.3 mL) was added dropwise, under an argon atmosphere, to a solution of amine **12** (6.5 g, 27.9 mmol) in acetic acid (325 mL). After 2.5 h of stirring at room temperature, the solvent was removed *in vacuo*. The excess of acetic acid was co-evaporated with heptane to afford the expected compound **13** (10.1 g, 99% yield) as a beige solid, mp = 163–165 °C. IR (cm^−1^) ν*_max_* 3327, 2826, 2605, 2566, 2236, 1955, 1716, 1610, 1561, 1496, 1481, 1398, 1369, 1280, 1238, 1193, 1153, 1059, 963, 906, 838; ^1^H-NMR (DMSO-*d*_6_) δ 9.05 (s, 1H, NH), 7.11 (d, 1H, *J* = 8.7 Hz), 7.05 (d, 1H, *J* = 8.7 Hz), 6.01 (s, 2H, NH_2_), 1.43 (s, 9H); ^13^C-NMR (DMSO-*d*_6_) δ 153.6, 144.1, 131.6, 126.9, 119.6, 116.1, 112.8, 107.8, 79.5, 28.1; HRMS calcd for C_12_H_15_N_3_O_2_Br [M+H]^+^: 312.0348, found 312.0354.

*tert-Butyl-3-bromo-4-(4-chloro-5H-1,2,3-dithiazol-5-ylideneamino)-2-cyanophenylcarbamate* (**14**). A suspension of **13** (5.0 g, 16.0 mmol), 4,5-dichloro-1,2,3-dithiazolium chloride (7.34 g, 35.2 mmol) in dichloromethane (100 mL) was stirred at room temperature under argon atmosphere. After 3 h of stirring, pyridine (5.7 mL, 70.5 mmol) was added and the mixture was stirred again for 1 h at room temperature. The resulting solution was concentrated under reduced pressure. The obtained crude residue was purified by flash chromatography (DCM-petroleum ether, 5:5) to afford the expected compound **14** (3.3 g, 46% yield) as an orange solid, mp = 140–150 °C (dec). IR (cm^−1^) ν*_max_* 3366, 2977, 2935, 2227, 1714, 1597, 1570, 1560, 1508, 1392, 1369, 1270, 1238, 1156, 1057, 971, 859, 846, 809; ^1^H-NMR (DMSO-*d*_6_) δ 9.64 (s, 1H, NH), 7.60 (d, 1H, *J* = 8.7 Hz), 7.55 (d, 1H, *J* = 8.7 Hz), 1.48 (s, 9H); ^13^C-NMR (DMSO-*d*_6_) δ 163.5, 152.9, 147.4, 146.0, 140.0, 126.1, 123.6, 117.7, 115.3, 112.2, 80.4, 27.9; HRMS calcd for C_14_H_13_N_4_O_2_S_2_BrCl [M+H]^+^: 446.9352, found 446.9340.

*6-Amino-2-bromo-3-(4-chloro-5H-1,2,3-dithiazol-5-ylideneamino)benzonitrile* (**15**). A mixture of carbamate **14** (3.3 g, 7.4 mmol) and acetic acid (100 mL) was heated at 118 °C under microwaves (800 W) for 2 h. After cooling, the resulting solution was concentrated under reduced pressure to give the desired compound **15** (2.8 g, quantitative yield) as an orange solid, mp = 188–198 °C (dec.). IR (cm^−1^) ν*_max_*/3421, 3340, 3231, 2220, 1701, 1647, 1596, 1575, 1473, 1405, 1291, 1251, 1192, 1137, 973, 869, 847, 804; ^1^H-NMR (DMSO-*d*_6_) δ 7.36 (d, 1H, *J* = 9.0 Hz), 6.91 (d, 1H, *J* = 9.0 Hz), 6.55 (s, 2H, NH_2_); ^13^C-NMR (DMSO-*d*_6_) δ 159.6, 151.7, 146.7, 137.8, 124.4, 119.0, 116.4, 115.7, 97.2; HRMS calcd for C_9_H_5_N_4_S_2_BrCl [M+H]^+^): 346.8828, found 346.8846.

*6-Aminobenzo[d]thiazole-2,7-dicarbonitrile* (**16**). A suspension of imine **15** (2.5 g, 7.2 mmol), copper(I) iodide (2.7 g, 14.4 mmol) in pyridine (50 mL) was heated at 130 °C under microwaves (400 W) for 20 min. After cooling, the mixture was dissolved in EtOAc and washed with a sodium thiosulfate solution. The organic layer was dried over MgSO_4_, and the solvent was removed *in vacuo*. The crude residue was purified by flash chromatography (DCM–EtOAc, 9:1) to afford the expected compound **16** (0.96 g, 67% yield) as a pale brown solid; mp = 248 °C. IR (KBr) ν*_max_*/cm^−1^ 3433, 3350, 3250, 2225, 1653, 1593, 1487, 1451, 1415, 1330, 1290, 1206, 1161, 1128, 821; ^1^H-NMR (DMSO-*d*_6_) δ 8.11 (d, 1H, *J* = 9.3 Hz), 7.31 (s, 2H, NH_2_), 7.10 (d, 1H, *J* = 9.3 Hz); ^13^C-NMR (DMSO-*d*_6_) δ 154.6, 143.7, 141.6, 131.5, 130.9, 119.4, 116.9, 114.3, 83.6; HRMS calcd for C_9_H_3_N_4_S (M−H)^−^: 199.0078, found 199.0076.

*(E)-N'-(2,7-Dicyanobenzo[d]thiazol-6-yl)-N,N-dimethylformimidamide* (**17**). A suspension of **16** (0.47 g, 2.34 mmol) in dimethylformamide dimethyl acetal (6 mL) was heated at 70 °C under microwaves (600 W) during 2 min. After cooling, the brown precipitate formed was filtered, washed with Et_2_O and dried to afford the expected compound **17** (0.51 g, 86% yield) as a brown solid; mp = 185–187 °C. IR (cm^−1^) ν*_max_* 2932, 2901, 2224, 1622, 1566, 1500, 1450, 1410, 1387, 1368, 1272, 1229, 1173, 1099, 1058, 995, 964, 928, 874, 819; ^1^H-NMR (DMSO-*d*_6_) δ 8.33 (d, 2H, *J* = 9.3 Hz), 7.62 (d, 1H, *J* = 9.3 Hz), 3.15 (s, 3H), 3.07 (s, 3H); ^13^C-NMR (DMSO-*d*_6_) δ 157.3, 156.5, 145.9, 140.2, 133.0, 129.3, 120.3, 116.6, 113.3, 96.4, 34.3; HRMS calcd for C_12_H_10_N_5_S [M+H]^+^: 256.0657, found 256.0644.

#### 3.2.6. Synthesis of Thiazolo[5,4-*f*]quinazoline-2-carbonitriles **7**–**10**

##### A-General Procedure from **6a**–**d**

A suspension of imine **6a**, **6c** and **6d** (1 mmol), copper iodide (2 mmol) in pyridine (4 mL) was heated at 120 °C (600 W) for 20 min. After cooling, the mixture was dissolved in ethyl acetate, washed with sodium thiosulfate solution. The organic layer was dried over MgSO_4_, and the solvent was removed *in vacuo*. The crude residue was purified by flash chromatography (DCM–EtOAc, 8:2) to afford the expected compounds **7**, **9** and **10**.

##### B-General Procedure from **17**

A mixture of (*E*)-*N*'-(2,7-dicyanobenzo[*d*]thiazol-6-yl)-*N,N*-dimethylformimidamide (**17**, 0.05 g, 0.19 mmol) and the appropriate amine (0.29 mmol, 1.5 eq) in acetic acid (2 mL) was heated under microwaves at 118 °C (600 W). On completion (followed by TLC), the reaction was cooled to ambient temperature. The solvent was removed *in vacuo* and the crude residue was purified by flash chromatography to afford the expected compounds **7**–**10**.

*9-(4-Methoxyphenylamino)thiazolo[5,4-f]quinazoline-2-carbonitrile* (**7**). Orange solid (yield: (A) 24%; (B) 99%); mp > 260 °C. IR IR (cm^−1^) ν*_max_* 3346, 2977, 2361, 2227, 1644, 1609, 1581, 1503, 1460, 1377, 1354, 1303, 1239, 1164, 1129, 1051, 1032, 975, 829; ^1^H-NMR (DMSO-*d*_6_) δ 8.44 (s, 1H), 7.95 (d, 1H, *J* = 9.0 Hz), 7.70 (d, 1H, *J* = 9.0 Hz), 7.45 (d, 2H, *J* = 9.0 Hz), 6.99 (d, 2H, *J* = 9.0 Hz), 3.76 (s, 3H); HRMS calcd for C_17_H_12_N_5_OS [M+H]^+^: 334.0763, found 334.0758.

*9-(Benzo[d][1,3]dioxol-5-ylamino)thiazolo[5,4-f]quinazoline-2-carbonitrile* (**8**). Orange solid (yield: (B) 95%); mp > 260 °C. IR (cm^−1^) ν*_max_* 2894, 2226, 1734, 1706, 1645, 1609, 1581, 1555, 1499, 1471, 1377, 1354, 1304, 1264, 1236 KBr, 1211, 1188, 1162, 1125, 1086, 1037, 972, 938, 924, 859, 829, 809; ^1^H-NMR (DMSO-*d*_6_) δ 8.51 (d, 1H, *J* = 9.0 Hz), 8.14 (m, 1H), 7.76 (m, 1H), 6.94 (d, 2H, *J* = 9.0 Hz), 6.72 (m, 1H), 6.02 (s, 2H); HRMS calcd for C_17_H_9_N_5_O_2_S [M+H]^+^: 348.0555, found 348.0566.

*9-(4-Bromo-2-fluorophenylamino)thiazolo[5,4-f]quinazoline-2-carbonitrile* (**9**). Brown solid (yield: (A) 30%; (B) 70%); mp > 260 °C. IR (cm^−1^) ν*_max_* 3325, 3053, 2230, 1649, 1614, 1582, 1556, 1499, 1462, 1380, 1351, 1250, 1154, 1132, 1052, 969, 904, 875, 817; ^19^F-NMR (DMSO-*d*_6_) δ −119.9; ^1^H-NMR (DMSO-*d*_6_) δ 8.55 (d, 1H, *J* = 9.0 Hz), 8.26 (d, 1H, *J* = 9.0 Hz), 7.78 (m, 1H), 7.55–7.52 (m, 1H), 7.38–7.25 (m, 2H), HRMS calcd for C_16_H_8_N_5_SBrF [M+H]^+^: 399.9668, found 399.9662.

*9-(3-Chloro-4-fluorophenylamino)thiazolo[5,4-f]quinazoline-2-carbonitrile* (**10**). Yellow solid (yield: (A) 64%; (B) 77%); mp = 252 °C. IR (cm^−1^) ν*_max_* 3456, 3015, 2970, 2946, 2229, 1642, 1441, 1153, 1129, 1051, 968, 903, 817, 774, 695; ^19^F-NMR (DMSO-*d*_6_) δ −123.8; ^1^H-NMR (DMSO-*d*_6_) δ 8.43 (d, 1H, *J* = 9.0 Hz), 8.30 (s, 1H), 7.62 (m, 2H), 7.34 (m, 2H); HRMS calcd for C_16_H_8_N_5_SClF [M+H]^+^: 356.0156, found 356.0167.

#### 3.2.7. Synthesis of Carboximidamides **7a**–**g**, **8a**–**g**, **9a**–**g** and **10a**–**g**

General procedure: A stirred mixture of carbonitrile **16** (1 mmol) and appropriate amine (1.2 mmol) in dry THF (7 mL) under argon was stirred overnight at room temperature. The solvent was removed *in vacuo* and the crude residue purified by flash chromatography to afford the expected carboximidamides.

*9-(4-Methoxyphenylamino)-N-(2-morpholinoethyl)thiazolo[5,4-f]quinazoline-2-carboximidamide* (**7a**). Prepared from carbonitrile **7** and *N*-aminoethylmorpholine. Flash chromatography eluent (DCM–MeOH, 5:5). Yellow solid (yield: 41%); mp = 130–132 °C. IR (cm^−1^) ν*_max_* 3362, 3175, 2920, 2852, 1643, 1567, 1504, 1469, 1386, 1234, 1111, 1032, 964, 836; ^1^H-NMR (MeOD-*d*_4_) δ 8.37 (d, 1H, *J* = 9.0 Hz), 8.11 (s, 1H), 7.75 (d, 1H, *J* = 9.0 Hz), 7.26 (d, 2H, *J* = 9.0 Hz), 7.01 (d, 2H, *J* = 9.0 Hz), 3.82 (s, 3H), 3.73 (m, 4H), 3.48 (t, 2H, *J* = 7.0 Hz), 2.76 (t, 2H, *J* = 7.0 Hz), 2.62 (m, 4H); HRMS calcd for C_23_H_26_N_7_O_2_S [M+H]^+^: 464.1869, found 464.1874.

*9-(4-Methoxyphenylamino)-N-(2-(piperidin-1-yl)ethyl)thiazolo[5,4-f]quinazoline-2-carboximidamide* (**7b**). Prepared from carbonitrile **7** and *N*-aminoethylpiperidine. Flash chromatography eluent (DCM–MeOH, 3:7). Yellow solid (yield: 43%); mp = 132–134 °C. IR (cm^−1^) ν*_max_* 2933, 2852, 1640, 1613, 1572, 1507, 1376, 1349, 1302, 1283, 1237, 1155, 1124, 1035, 962, 833; ^1^H-NMR (MeOD-*d*_4_) δ 8.34 (d, 1H, *J* = 9.0 Hz), 8.08 (s, 1H), 7.73 (d, 1H, *J* = 9.0 Hz), 7.23 (d, 2H, *J* = 9.0 Hz), 6.99 (d, 2H, *J* = 9.0 Hz), 3.82 (s, 3H), 3.49 (t, 2H, *J* = 7.0 Hz), 2.73 (t, 2H, *J* = 7.0 Hz), 2.56 (m, 4H), 1.66 (m, 4H), 1.52 (m, 2H); HRMS calcd for C_24_H_28_N_7_OS [M+H]^+^: 462.2076, found 462.2098.

*9-(4-Methoxyphenylamino)-N-(2-(pyrrolidin-1-yl)ethyl)thiazolo[5,4-f]quinazoline-2-carboximidamide* (**7c**). Prepared from carbonitrile **7** and *N*-aminoethylpyrrolidine. Flash chromatography eluent (DCM–MeOH, 5:5). Orange solid (yield: 47%); mp = 134–136 °C. IR (cm^−1^) ν*_max_* 2957, 2798, 1643, 1615, 1573, 1504, 1379, 1341, 1284, 1237, 1147, 1086, 1033, 962, 880, 833; ^1^H-NMR (MeOD-*d*_4_) δ 8.34 (d, 1H, *J* = 9.0 Hz), 8.08 (s, 1H), 7.73 (d, 1H, *J* = 9.0 Hz), 7.23 (d, 2H, *J* = 9.0 Hz), 6.99 (d, 2H, *J* = 9.0 Hz), 3.82 (s, 3H), 3.49 (t, 2H, *J* = 7.0 Hz), 2.88 (t, 2H, *J* = 7.0 Hz), 2.67 (m, 4H), 1.85 (m, 4H); HRMS calcd for C_23_H_26_N_7_OS [M+H]^+^: 448.1920, found 448.1926.

*N-(2-(Dimethylamino)ethyl)-9-(4-methoxyphenylamino)thiazolo[5,4-f]quinazoline-2-carboximidamide* (**7d**). Prepared from carbonitrile **7** and 2-dimethylaminoethylamine. Flash chromatography eluent (DCM–MeOH, 5:5). Yellow solid (yield: 53%); mp = 154–156 °C. IR (cm^−1^) ν*_max_* 2945, 2827, 2773, 1643, 1614, 1572, 1505, 1464, 1379, 1341, 1237, 1177, 1033, 962, 832; ^1^H-NMR (MeOD-*d*_4_) δ 8.30 (d, 1H, *J* = 9.0 Hz), 8.06 (s, 1H), 7.69 (d, 1H, *J* = 9.0 Hz), 7.22 (d, 2H, *J* = 9.0 Hz), 6.97 (d, 2H, *J* = 9.0 Hz), 3.81 (s, 3H), 3.43 (t, 2H, *J* = 7.0 Hz), 2.70 (t, 2H, *J* = 7.0 Hz), 2.34 (s, 6H); HRMS calcd for C_21_H_24_N_7_OS [M+H]^+^: 422.1763, found 422.1766.

*N-(2-(Diethylamino)ethyl)-9-(4-methoxyphenylamino)thiazolo[5,4-f]quinazoline-2-carboximidamide* (**7e**). Prepared from carbonitrile **7** and diethylethylenediamine. Flash chromatography eluent (DCM–MeOH, 5:5). Yellow solid (yield: 50%); mp = 103–105 °C. IR (cm^−1^) ν*_max_* 2967, 2832, 1641, 1614, 1572, 1507, 1378, 1341, 1285, 1237, 1178, 1086, 1034, 962, 834; ^1^H-NMR (MeOD-*d*_4_) δ 8.35 (d, 1H, *J* = 9.0 Hz), 8.08 (s, 1H), 7.74 (d, 1H, *J* = 9.0 Hz), 7.23 (d, 2H, *J* = 9.0 Hz), 7.01 (d, 2H, *J* = 9.0 Hz), 3.82 (s, 3H), 3.43 (t, 2H, *J* = 7.0 Hz), 2.86 (t, 2H, *J* = 7.0 Hz), 2.71 (q, 4H, *J* = 7.0 Hz), 1.11 (t, 6H, *J* = 7.0 Hz); HRMS calcd for C_23_H_28_N_7_OS [M+H]^+^: 450.2076, found 450.2058.

*N-Benzyl-9-(4-methoxyphenylamino)thiazolo[5,4-f]quinazoline-2-carboximidamide* (**7f**). Prepared from carbonitrile **7** and benzylamine. Flash chromatography eluent (DCM–EtOAc, 2:8). Yellow solid (yield: 28%); mp = 128–130 °C. IR (cm^−1^) ν*_max_* 3362, 3028, 2832, 1640, 1598, 1572, 1509, 1377, 1356, 1299, 1238, 1177, 1084, 1030, 962, 833; ^1^H-NMR (MeOD-*d*_4_) δ 8.34 (d, 1H, *J* = 9.0 Hz), 8.08 (s, 1H), 7.73 (d, 1H, *J* = 9.0 Hz), 7.35 (m, 4H), 7.26 (m, 3H), 6.99 (d, 2H, *J* = 9.0 Hz), 4.55 (s, 2H), 3.83 (s, 3H); HRMS calcd for C_24_H_21_N_6_OS [M+H]^+^: 441.1498, found 441.1507.

*9-(4-Methoxyphenylamino)-N,N-dimethylthiazolo[5,4-f]quinazoline-2-carboximidamide* (**7g**). Prepared from carbonitrile **7** and dimethylamine. Flash chromatography eluent (DCM–MeOH, 5:5). Pale yellow solid (yield: 67%); mp = 150–152 °C. IR (KBr) ν*_max_*/cm^−1^ 3139, 2924, 1681, 1644, 1571, 1509, 1383, 1347, 1286, 1237, 1176, 1031, 966, 834; ^1^H-NMR (MeOD-*d*_4_) δ 8.15 (d, 1H, *J* = 9.0 Hz), 7.84 (s, 1H), 7.53 (d, 1H, *J* = 9.0 Hz), 7.08 (d, 2H, *J* = 9.0 Hz), 6.89 (d, 2H, *J* = 9.0 Hz), 3.77 (s, 3H), 3.13 (s, 6H); HRMS calcd for C_19_H_19_N_6_OS [M+H]^+^: 379.1341, found 379.1333.

*9-(Benzo[d][1,3]dioxol-5-ylamino)-N-(2-morpholinoethyl)thiazolo[5,4-f]quinazoline-2-carboximidamide* (**8a**). Prepared from carbonitrile **8** and *N*-aminoethylmorpholine. Flash chromatography eluent (DCM–MeOH, 5:5). Yellow solid (yield: 41%); mp = 168–170 °C. IR (cm^−1^) ν*_max_* 3324, 2965, 2901, 2861, 2812, 1641, 1616, 1587, 1503, 1468, 1382, 1353, 1304, 1271, 1232, 1180, 1148, 1114, 1067, 1036, 969, 935, 839; ^1^H-NMR (MeOD-*d*_4_) δ 8.33 (d, 1H, *J* = 9.0 Hz), 7.96 (s, 1H), 7.71 (d, 1H, *J* = 9.0 Hz), 6.87 (d, 1H, *J* = 9.0 Hz), 6.74 (s, 1H), 6.64 (d, 1H, *J* = 9.0 Hz), 5.96 (s, 2H), 3.74 (m, 4H), 3.55 (t, 2H, *J* = 7.0 Hz), 2.75 (t, 2H, *J* = 7.0 Hz), 2.61 (m, 4H); HRMS calcd for C_23_H_24_N_7_O_3_S [M+H]^+^: 478.1661, found 478.1649.

*9-(Benzo[d][1,3]dioxol-5-ylamino)-N-(2-(piperidin-1-yl)ethyl)thiazolo[5,4-f]quinazoline-2-carboximi-damide* (**8b**). Prepared from carbonitrile **8** and *N*-aminoethylpiperidine. Flash chromatography eluent (DCM–MeOH, 3:7). Yellow solid (yield: 34%); mp = 170–172 °C. IR (cm^−1^) ν*_max_* 3350, 2936, 2773, 2482, 2061, 1641, 1613, 1585, 1503, 1462, 1374, 1337, 1304, 1229, 1176, 1141, 1121, 1037, 983, 962, 931, 855, 832; ^1^H-NMR (MeOD-*d*_4_) δ 8.33 (d, 1H, *J* = 9.0 Hz), 7.89 (s, 1H), 7.69 (d, 1H, *J* = 9.0 Hz), 6.86 (d, 1H, *J* = 9.0 Hz), 6.75 (s, 1H), 6.63 (d, 1H, *J* = 9.0 Hz), 5.99 (s, 2H), 3.46 (t, 2H, *J* = 7.0 Hz), 2.71 (t, 2H, *J* = 7.0 Hz), 2.57 (m, 4H), 1.64 (m, 4H), 1.50 (m, 2H); HRMS calcd for C_24_H_26_N_7_O_2_S [M+H]^+^: 476.1869, found 476.1883.

*9-(Benzo[d][1,3]dioxol-5-ylamino)-N-(2-(pyrrolidin-1-yl)ethyl)thiazolo[5,4-f]quinazoline-2-carboxi-midamide* (**8c**). Prepared from carbonitrile **8** and *N*-aminoethylpyrrolidine. Flash chromatography eluent (DCM–MeOH, 5:5). Yellow solid (yield: 48%); mp = 172–174 °C. IR (cm^−1^) ν*_max_* 2906, 2803, 1643, 1614, 1573, 1500, 1467, 1377, 1347, 1305, 1265, 1228, 1175, 1121, 1036, 966, 933, 833; ^1^H-NMR (MeOD-*d*_4_) δ 8.27 (d, 1H, *J* = 9.0 Hz), 7.90 (s, 1H), 7.65 (d, 1H, *J* = 9.0 Hz), 6.86 (d, 1H, *J* = 9.0 Hz), 6.75 (s, 1H), 6.63 (d, 1H, *J* = 9.0 Hz), 5.97 (s, 2H), 3.46 (t, 2H, *J* = 7.0 Hz), 2.85 (t, 2H, *J* = 7.0 Hz), 2.67 (m, 4H), 1.83 (m, 4H); HRMS calcd for C_23_H_24_N_7_O_2_S [M+H]^+^: 462.1712, found 462.1736.

*9-(Benzo[d][1,3]dioxol-5-ylamino)-N-(2-(dimethylamino)ethyl)thiazolo[5,4-f]quinazoline-2-carboxi-midamide* (**8d**). Prepared from carbonitrile **8** and 2-dimethylaminoethylamine. Flash chromatography eluent (DCM–MeOH, 5:5). Yellow solid (yield: 10%); mp > 260 °C. IR (cm^−1^) ν*_max_* 3391, 2923, 2852, 1643, 1615, 1538, 1498, 1467, 1383, 1346, 1302, 1263, 1229, 1178, 1118, 1035, 965, 933, 856, 835; ^1^H-NMR (MeOD-*d*_4_) δ 8.17 (d, 1H, *J* = 9.0 Hz), 7.90 (s, 1H), 7.56 (d, 1H, *J* = 9.0 Hz), 6.86 (d, 1H, *J* = 9.0 Hz), 6.75 (s, 1H), 6.63 (d, 1H, *J* = 9.0 Hz), 5.91 (s, 2H), 3.43 (t, 2H, *J* = 7.0 Hz), 2.69 (t, 2H, *J* = 7.0 Hz), 2.33 (s, 6H); HRMS calcd for C_21_H_22_N_7_O_2_S [M+H]^+^: 436.1556, found 436.1549.

*9-(Benzo[d][1,3]dioxol-5-ylamino)-N-(2-(diethylamino)ethyl)thiazolo[5,4-f]quinazoline-2-carboxi-midamide* (**8e**). Prepared from carbonitrile **8** and diethylethylenediamine. Flash chromatography eluent (DCM–MeOH, 5:5). Yellow solid (yield: 44%); mp = 144–146 °C. IR (cm^−1^) ν*_max_* 3333, 2970, 2920, 2832, 1641, 1614, 1585, 1502, 1463, 1421, 1378, 1348, 1303, 1231, 1179, 1145, 1122, 1088, 1037, 965, 933, 858, 831; ^1^H-NMR (MeOD-*d*_4_) δ 8.23 (d, 1H, *J* = 9.0 Hz), 7.89 (s, 1H), 7.62 (d, 1H, *J* = 9.0 Hz), 6.86 (d, 1H, *J* = 9.0 Hz), 6.75 (s, 1H), 6.63 (d, 1H, *J* = 9.0 Hz), 5.94 (s, 2H), 3.42 (t, 2H, *J* = 7.0 Hz), 2.83 (t, 2H, *J* = 7.0 Hz), 2.68 (q, 4H, *J* = 7.0 Hz), 1.10 (t, 6H, *J* = 7.0 Hz); HRMS calcd for C_23_H_26_N_7_O_2_S [M+H]^+^: 464.1869, found 464.1896.

*9-(Benzo[d][1,3]dioxol-5-ylamino)-N-benzylthiazolo[5,4-f]quinazoline-2-carboximidamide* (**8f**). Prepared from carbonitrile **8** and benzylamine. Flash chromatography eluent (DCM–EtOAc, 2:8). Yellow solid (yield: 21%); mp > 260 °C. IR (cm^−1^) ν*_max_* 2886, 2447, 1641, 1597, 1497, 1466, 1413, 1384, 1353, 1307, 1266, 1229, 1178, 1122, 1105, 1036, 967, 934, 861, 833; ^1^H-NMR (MeOD-*d*_4_) δ 8.27 (d, 1H, *J* = 9.0 Hz), 7.90 (s, 1H), 7.65 (d, 1H, *J* = 9.0 Hz), 6.86 (m, 4H), 6.75 (m, 3H), 6.63 (d, 1H, *J* = 9.0 Hz), 5.97 (s, 2H), 4.58 (s, 2H); HRMS calcd for C_24_H_19_N_6_O_2_S [M+H]^+^: 455.1290, found 455.1287.

*9-(4-Bromo-2-fluorophenylamino)-N-(2-morpholinoethyl)thiazolo[5,4-f]quinazoline-2-carboximid-amide* (**9a**). Prepared from carbonitrile **9** and *N*-aminoethylmorpholine. Flash chromatography eluent (DCM–MeOH, 5:5). Yellow solid (yield: 85%); mp = 190–192 °C. IR (cm^−1^) ν*_max_* 3374, 2975, 2361, 1645, 1585, 1381, 1265, 1227, 1090, 1053, 973, 916, 882, 825; ^19^F-NMR (DMSO-*d*_6_) δ −120.0; ^1^H-NMR (DMSO-*d*_6_) δ 8.52 (d, 1H, *J* = 9.0 Hz), 8.26 (m, 1H), 7.78 (m, 1H), 7.57 (m, 1H), 7.36 (m, 1H), 7.16 (m, 1H), 3.66 (t, 4H, *J* = 4.5 Hz), 3.48–3.42 (m, 2H), 2.69 (t, 2H, *J* = 6.0 Hz), 2.61 (m, 4H); HRMS calcd for C_22_H_22_N_7_OSBrF [M+H]^+^: 530.0774, found 530.0782.

*9-(4-Bromo-2-fluorophenylamino)-N-(2-(piperidin-1-yl)ethyl)thiazolo[5,4-f]quinazoline-2-carboxi-midamide* (**9b**). Prepared from carbonitrile **9** and *N*-aminoethylpiperidine. Flash chromatography eluent (DCM–MeOH, 5:5). Yellow solid (yield: 72%); mp = 142–144 °C. IR (cm^−1^) ν*_max_* 3340, 2975, 2930, 2361, 1615, 1561, 1476, 1380, 1348, 1262, 1197, 1153, 1114, 1052, 881, 821; ^19^F-NMR (CDCl_3_) δ −123.8; ^1^H-NMR (CDCl_3_) δ 8.25 (d, 1H, *J* = 9.0 Hz), 8.09 (s, 1H), 7.55 (d, 1H, *J* = 9.0 Hz), 7.50–7.45 (m, 1H), 7.29 (t, 1H, *J* = 9.0 Hz), 7.12–7.06 (m, 1H), 3.54 (m, 2H), 2.67 (t, 2H, *J* = 5.7 Hz), 2.54 (m, 4H), 1.66–1.60 (m, 4H), 1.49 (m, 2H); HRMS calcd for C_23_H_24_N_7_SBrF [M+H]^+^: 528.0981, found 528.0986.

*9-(4-Bromo-2-fluorophenylamino)-N-(2-(pyrrolidin-1-yl)ethyl)thiazolo[5,4-f]quinazoline-2-carboxi-midamide* (**9c**). Prepared from carbonitrile **9** and *N*-aminoethylpyrrolidine. Flash chromatography eluent (DCM–MeOH, 5:5). Orange solid (yield: 68%); mp = 138–140 °C. IR (cm^−1^) ν*_max_* 2968, 2361, 1563, 1476, 1380, 1262, 1223, 1067, 965, 880, 822; ^19^F-NMR (MeOD-*d*_4_) δ −123.6; ^1^H-NMR (MeOD-*d*_4_) δ 8.29 (d, 1H, *J* = 9.0 Hz), 7.89 (s, 1H), 7.63 (d, 1H, *J* = 9.0 Hz), 7.35–7.29 (m, 2H), 7.09 (t, 1H, *J* = 9.0 Hz), 3.46 (t, 2H, *J* = 6.6 Hz), 2.87 (t, 2H, *J* = 6.6 Hz), 2.69 (m, 4H), 1.89 (m, 4H); HRMS calcd for C_22_H_22_N_7_SBrF [M+H]^+^: 514.0825, found 514.0825.

*9-(4-Bromo-2-fluorophenylamino)-N-(2-(dimethylamino)ethyl)thiazolo[5,4-f]quinazoline-2-carboxi-midamide* (**9d**). Prepared from carbonitrile **9** and 2-dimethylaminoethylamine. Flash chromatography eluent (DCM–MeOH, 5:5). Orange solid (yield: 64%); mp = 128–130 °C. IR (cm^−1^) ν*_max_* 3274, 3058, 2940, 2861, 2822, 2773, 1618, 1560, 1478, 1380, 1341, 1262, 1197, 1155, 1112, 1038, 965, 881, 823; ^19^F-NMR (MeOD-*d*_4_ ) δ −122.3; ^1^H-NMR (MeOD-*d*_4_) δ 8.33 (d, 1H, *J* = 9.0 Hz), 7.92 (s, 1H), 7.68 (d, 1H, *J* = 9.0 Hz), 7.41–7.36 (m, 2H), 7.15 (t, 1H, *J* = 9.0 Hz), 3.46 (t, 2H, *J* = 6.6 Hz), 2.76 (t, 2H, *J* = 6.6 Hz), 2.37 (s, 6H); HRMS calcd for C_20_H_20_N_7_SBrF [M+H]^+^: 488.0668, found 488.0688.

*9-(4-Bromo-2-fluorophenylamino)-N-(2-(diethylamino)ethyl)thiazolo[5,4-f]quinazoline-2-carboxi-midamide* (**9e**). Prepared from carbonitrile **9** and diethylethylenediamine. Flash chromatography eluent (DCM–MeOH, 5:5). Orange solid (yield: 86%); mp = 88–100 °C. IR (cm^−1^) ν*_max_* 2965, 2360, 1793, 1619, 1520, 1477, 1378, 1265, 1198, 1067, 966, 880, 822; ^19^F-NMR (MeOD-*d*_4_) δ −122.1; ^1^H-NMR (MeOD-*d*_4_) δ 8.30 (d, 1H, *J* = 9.0 Hz), 7.93 (s, 1H), 7.66 (d, 1H, *J* = 9.0 Hz), 7.38–7.33 (m, 2H), 7.13 (t, 1H, *J* = 9.0 Hz), 3.58 (t, 2H, *J* = 6.6 Hz), 2.82 (t, 2H, *J* = 6.6 Hz), 2.68 (m, 4H), 1.09 (m, 6H); HRMS calcd for C_22_H_24_N_7_SBrF [M+H]^+^: 516.0981, found 516.0988.

*N-Benzyl-9-(4-bromo-2-fluorophenylamino)thiazolo[5,4-f]quinazoline-2-carboximidamide* (**9f**). Prepared from carbonitrile **9** and benzylamine. Flash chromatography eluent (DCM–EtOAc, 2:8). Yellow solid (yield: 68%); mp = 130–132 °C. IR (cm^−1^) ν*_max_* 2964, 2903, 2360, 1815, 1614, 1477, 1379, 1262, 1153, 1113, 1069, 966, 880, 821; ^19^F-NMR (MeOD-*d*_4_) δ −122.2; ^1^H-NMR (MeOD-*d*_4_) δ 8.35 (d, 1H, *J* = 9.0 Hz), 7.92 (s, 1H), 7.69 (d, 1H, *J* = 9.0 Hz), 7.43 (m, 3H), 7.39–7.30 (m, 3H), 7.25 (m, 1H), 7.15 (t, 1H, *J* = 9.0 Hz), 4.53 (s, 2H); HRMS calcd for C_23_H_17_N_6_SBrF [M+H]^+^: 507.0403, found 507.0412.

*9-(4-Bromo-2-fluorophenylamino)-N-(2-(dimethylamino)ethyl)thiazolo[5,4-f]quinazoline-2-carboxi-midamide* (**9g**). Prepared from carbonitrile **9** and dimethylamine. Flash chromatography eluent (DCM–MeOH, 5:5). Orange solid (yield: 40%); mp = 222–224 °C. IR (cm^−1^) ν*_max_* 3270, 3154, 3061, 2923, 1634, 1584, 1519, 1492, 1410, 1346, 1291, 1226, 1201, 1152, 1116, 1049, 964, 881, 863, 831; ^19^F-NMR (MeOD-*d*_4_) δ −121.6; ^1^H-NMR (MeOD-*d*_4_) δ 8.18 (d, 1H, *J* = 9.0 Hz), 7.87 (s, 1H), 7.53 (d, 1H, *J* = 9.0 Hz), 7.28–7.19 (m, 2H), 7.12 (t, 1H, *J* = 9.0 Hz), 3.11 (s, 6H); HRMS calcd for C_18_H_15_N_6_SBrF [M+H]^+^: 445.0246, found 445.0264.

*9-(3-Chloro-4-fluorophenylamino)-N-(2-morpholinoethyl)thiazolo[5,4-f]quinazoline-2-carboximid-amide* (**10a**). Prepared from carbonitrile **10** and *N*-aminoethylmorpholine. Flash chromatography eluent (DCM–MeOH, 5:5). Yellow solid (yield: 71%); mp = 158–160 °C. IR (cm^−1^) ν*_max_* 2956, 2816, 1797, 1614, 1561, 1485, 1256, 1199, 1113, 1069, 966, 916, 816; ^19^F-NMR (DMSO-*d*_6_) δ −119.1; ^1^H-NMR (DMSO-*d*_6_) δ 8.87 (s, 1H, NH), 8.43 (d, 1H, *J* = 7.2 Hz), 8.03 (m, 1H), 7.90 (m, 1H), 7.52 (m, 1H), 7.39 (m, 1H), 7.18 (m, 1H), 3.78–3.75 (m, 4H), 3.52 (m, 2H), 2.76–2.72 (m, 2H), 2.60 (m, 4H); HRMS calcd for C_22_H_22_N_7_OSClF [M+H]^+^: 486.1279, found 486.1292.

*9-(3-Chloro-4-fluorophenylamino)-N-(2-(piperidin-1-yl)ethyl)thiazolo[5,4-f]quinazoline-2-carboxi-midamide* (**10b**). Prepared from carbonitrile **10** and *N*-aminoethylpiperidine. Flash chromatography eluent (DCM–MeOH, 3:7). Orange solid (yield: 82%); mp = 146–148 °C. IR (cm^−1^) ν*_max_* 2928, 2361, 1572, 1483, 1380, 1255, 1201, 1121, 1086, 1051, 964, 879, 818; ^19^F-NMR (CDCl_3_) δ ‒123.8; ^1^H-NMR (CDCl_3_) δ 8.62 (s, 1H), 8.35 (d, 1H, *J* = 9.0 Hz), 7.90 (d, 1H, *J* = 9.0 Hz), 7.70 (m, 1H), 7.32 (m, 1H), 7.12 (t, 1H, *J* = 9.0 Hz), 3.50 (m, 2H), 2.63 (t, 2H, *J* = 5.7 Hz), 2.49 (m, 4H), 1.63–1.58 (m, 4H), 1.48 (m, 2H); HRMS calcd for C_23_H_24_N_7_SClF [M + H]^+^: 484.1486, found 484.1501.

*9-(3-Chloro-4-fluorophenylamino)-N-(2-(pyrrolidin-1-yl)ethyl)thiazolo[5,4-f]quinazoline-2-carboxi-midamide* (**10c**). Prepared from carbonitrile **10** and *N*-aminoethylpyrrolidine. Flash chromatography eluent (DCM–MeOH, 5:5). Orange solid (yield: 34%); mp = 166–168 °C. IR (cm^−1^) ν*_max_* 3381, 3146, 2965, 2803, 1641, 1617, 1562, 1486, 1383, 1341, 1253, 1200, 1127, 1050, 965, 876, 816; ^19^F-NMR (MeOD-*d*_4_) δ −125.6; ^1^H-NMR (MeOD-*d*_4_) δ 8.22 (d, 1H, *J* = 9.0 Hz), 7.95 (s, 1H), 7.59 (d, 1H, *J* = 9.0 Hz), 7.31 (m, 1H), 7.18 (t, 1H, *J* = 9.0 Hz), 7.15–7.12 (m, 1H), 3.45 (m, 2H), 2.85 (m, 2H), 2.67 (m, 4H), 1.83 (m, 4H); HRMS calcd for C_22_H_22_N_7_SClF [M+H]^+^: 470.1330, found 470.1340.

*9-(3-Chloro-4-fluorophenylamino)-N-(2-(dimethylamino)ethyl)thiazolo[5,4-f]quinazoline-2-carboxi-midamide* (**10d**). Prepared from carbonitrile **10** and 2-dimethylaminoethylamine. Flash chromatography eluent (DCM–MeOH, 5:5). Pale yellow solid (yield: 50%); mp = 172–174 °C. IR (cm^−1^) ν*_max_* 3224, 3038, 2950, 2861, 2824, 2773, 1618, 1560, 1488, 1386, 1323, 1254, 1195, 1127, 1086, 1052, 967, 816; ^19^F-NMR (MeOD-*d*_4_) δ −125.5; ^1^H-NMR (MeOD-*d*_4_) δ 8.34 (d, 1H, *J* = 9.0 Hz), 7.93 (s, 1H), 7.67 (d, 1H, *J* = 9.0 Hz), 7.29 (m, 1H), 7.18 (t, 1H, *J* = 9.0 Hz), 7.10–7.06 (m, 1H), 3.64 (t, 2H, *J* = 6.0 Hz), 3.27 (t, 2H, *J* = 6.0 Hz), 2.80 (s, 6H); HRMS calcd for C_20_H_20_N_7_SClF [M+H]^+^: 444.1173, found 444.1155.

*9-(3-Chloro-4-fluorophenylamino)-N-(2-(diethylamino)ethyl)thiazolo[5,4-f]quinazoline-2-carboxi-midamide* (**10e**). Prepared from carbonitrile **10** and diethylethylenediamine. Flash chromatography eluent (DCM–MeOH, 5:5). Orange solid (yield: 50%); mp = 140–142 °C. IR (cm^−1^) ν*_max_* 3295, 2969, 2812, 1671, 1618, 1560, 1489, 1386, 1346, 1254, 1196, 1127, 1052, 966, 817; ^19^F-NMR (MeOD-*d*_4_) δ −125.3; ^1^H-NMR (MeOD-*d*_4_) δ 8.29 (d, 1H, *J* = 9.0 Hz), 7.94 (s, 1H), 7.63 (d, 1H, *J* = 9.0 Hz), 7.29–7.20 (m, 2H), 7.09 (m, 1H), 3.60–3.56 (m, 2H), 2.90–2.83 (m, 2H), 2.77–2.74 (m, 4H), 1.17–1.06 (m, 6H); HRMS calcd for C_22_H_24_N_7_SClF [M+H]^+^: 472.1486, found 472.1502.

*N-Benzyl-9-(3-chloro-4-fluorophenylamino)thiazolo[5,4-f]quinazoline-2-carboximidamide* (**10f**). Prepared from carbonitrile **10** and benzylamine. Flash chromatography eluent (DCM–EtOAc, 2:8). Yellow solid (yield: 69%); mp = 230–232 °C. IR (cm^−1^) ν*_max_* 3057, 1725, 1639, 1490, 1377, 1341, 1252, 1202, 1151, 1121, 1086, 1050, 965, 818; ^19^F-NMR (282 MHz, MeOD-*d*_4_) δ −125.3; ^1^H-NMR (300 MHz, MeOD-*d*_4_) δ 8.29 (d, 1H, *J* = 9.0 Hz), 7.94 (s, 1H), 7.63 (d, 1H, *J* = 9.0 Hz), 7.43 (m, 2H), 7.35–7.17 (m, 5H), 7.10 (m, 1H), 4.53 (s, 2H); HRMS calcd for C_23_H_17_N_6_SClF [M+H]^+^: 463.0908, found 463.0916.

*9-(3-Chloro-4-fluorophenylamino)-N,N-dimethylthiazolo[5,4-f]quinazoline-2-carboximidamide* (**10g**). Prepared from carbonitrile **10** and dimethylamine. Flash chromatography eluent (DCM–MeOH, 5:5). Yellow solid (yield: 43%); mp > 260 °C. IR (KBr) ν*_max_*/cm^−1^ 3411, 3051, 1663, 1623, 1559, 1488, 1383, 1254, 1202, 1150, 1051, 966, 819; ^19^F-NMR (MeOD-*d*_4_) δ −125.9; ^1^H-NMR (MeOD-*d*_4_) δ 8.45 (d, 1H, *J* = 9.0 Hz), 7.98 (s, 1H), 7.75 (d, 1H, *J* = 9.0 Hz), 7.30–7.21 (m, 2H), 7.12 (t, 1H, *J* = 9.0 Hz), 3.39 (s, 6H); HRMS calcd for C_18_H_15_N_6_SClF [M+H]^+^: 401.0751, found 401.0742.

#### 3.2.8. Synthesis of Amides **7h**–**10h**

General procedure: a stirred mixture of carbonitriles **7**–**10** (0.13 mmol) and NaOH (2.5 N sol., 50 μL) in butanol (2.5 mL) was heated under microwaves at 117 °C (600 W) for 30 min. The solvent was removed *in vacuo* and the crude residue purified by flash chromatography (DCM–EtOAc, 5:5) to afford amides **7h**–**10h**.

*9-(4-Methoxyphenylamino)thiazolo[5,4-f]quinazoline-2-carboxamide* (**7h**). Orange solid (yield: 98%); mp = 213 °C. IR (cm^−1^) ν*_max_* 3409, 1691, 1638, 1600, 1572, 1509, 1431, 1380, 1349, 1325, 1301, 1237, 1177, 1123, 1085, 1032, 964, 835, 814; ^1^H-NMR (DMSO-*d*_6_) δ 8.37 (s, 1H), 8.24 (d, 1H, *J* = 9.0 Hz), 8.06 (s, 1H), 7.92 (s, 1H), 7.61 (d, 1H, *J* = 9.0 Hz), 7.31 (d, 2H, *J* = 9.0 Hz), 6.89 (d, 2H, *J* = 9.0 Hz), 3.74 (s, 3H); HRMS calcd for C_17_H_14_N_5_O_2_S [M+H]^+^: 352.0868, found 352.0879.

*9-(Benzo[d][1,3]dioxol-5-ylamino)thiazolo[5,4-f]quinazoline-2-carboxamide* (**8h**). Yellow solid (yield: 31%); mp > 260 °C. IR (KBr) ν*_max_*/cm^−1^ 3291, 2915, 1648, 1576, 1532, 1497, 1476, 1376, 1351, 1323, 1272, 1191, 1104, 1035, 965, 923, 830, 815; ^1^H-NMR (DMSO-*d*_6_) δ 8.05–7.98 (m, 2H), 7.49 (d, 1H, *J* = 2 Hz), 7.37–7.31 (m, 1H), 6.96 (dd, 1H, *J*_1_ = 2 Hz, *J*_2_ = 9 Hz), 6.73 (dd, 1H, *J*_1_ = 2 Hz, *J*_2_ = 9 Hz), 5.88 (s, 2H); HRMS calcd for C_17_H_12_N_5_O_3_S [M+H]^+^: 366.0661, found 366.0658.

*9-(4-Bromo-2-fluorophenylamino)thiazolo[5,4-f]quinazoline-2-carboxamide* (**9h**). Yellow solid (yield: 71%); mp > 260 °C. IR (KBr) ν*_max_*/cm^−1^ 1682, 1645, 1615, 1575, 1557, 1486, 1347, 1254, 1200, 1158, 1118, 1074, 993, 967, 941, 865, 819; ^19^F-NMR (DMSO-*d*_6_) δ −120.5; ^1^H-NMR (DMSO-*d*_6_) δ 8.47 (s, 1H), 8.41 (d, 1H, *J* = 9.0 Hz), 8.39 (s, 1H), 8.14 (s, 1H), 8.03 (d, 1H, *J* = 9.0 Hz), 7.53 (m, 1H), 7.36 (m, 1H), 7.21 (t, 1H, *J* = 9.0 Hz); HRMS calcd for C_16_H_10_N_5_OSBrF [M+H]^+^: 417.9769, found 417.9769.

*9-(3-Chloro-4-fluorophenylamino)thiazolo[5,4-f]quinazoline-2-carboxamide* (**10h**). Orange solid (yield: 98%); mp > 260 °C. IR (KBr) ν*_max_*/cm^−1^ 3453, 1684, 1624, 1601, 1576, 1534, 1506, 1487, 1376, 1348, 1282, 1260, 1209, 1124, 1085, 1057, 993, 969, 825, 810; ^19^F-NMR (DMSO-*d*_6_) δ −129.1; ^1^H-NMR (DMSO-*d*_6_) δ 8.26–8.21 (m, 2H), 8.12 (d, 1H, *J* = 9.0 Hz), 8.02–7.99 (m, 1H), 7.80 (s, 1H), 7.53 (d, 1H, *J* = 9.0 Hz), 7.47 (m, 1H), 7.18 (t, 1H, *J* = 9.0 Hz); HRMS calcd for C_16_H_10_N_5_OSClF [M+H]^+^: 374.0279, found 374.0280.

#### 3.2.9. Synthesis of Methylimidates **7i**–**10i**

General procedure: a stirred mixture of carbonitriles **7**–**10** (0.13 mmol) and NaOCH_3_ (0.5 M sol. in MeOH, 130 μL) in methanol (4 mL) was heated under microwaves at 65 °C (600 W) for 30 min. The solvent was removed *in vacuo* and the crude residue purified by flash chromatography (DCM–EtOAc) to afford imidates **7i**–**10i**.

*Methyl 9-(4-methoxyphenylamino)thiazolo[5,4-f]quinazoline-2-carbimidate* (**7i**). Purified by flash chromatography (DCM–EtOAc, 5:5) as orange solid (yield: 82%); mp = 240–242 °C. IR (cm^−1^); ν*_max_* 2833, 2354, 1644, 1614, 1567, 1504, 1439, 1397, 1373, 1349, 1325, 1286, 1240, 1215, 1181, 1155, 1104, 1069, 1033, 965, 935, 859, 832, 814; ^1^H-NMR (MeOD-*d*_4_) δ 8.09 (d, 1H, *J* = 9.0 Hz), 7.93 (s, 1H), 7.50 (d, 1H, *J* = 9.0 Hz), 7.14 (d, 2H, *J* = 9.0 Hz), 6.82 (d, 2H, *J* = 9.0 Hz), 3.98 (s, 3H), 3.74 (s, 3H); HRMS calcd for C_18_H_16_N_5_O_2_S [M+H]^+^: 366.1025, found 366.1034.

*Methyl 9-(benzo[d][1,3]dioxol-5-ylamino)thiazolo[5,4-f]quinazoline-2-carbimidate* (**8i**). Purified by flash chromatography (DCM–EtOAc, 5:5) as yellow solid (yield: 92%); mp = 230–232 °C. IR (cm^−1^) ν*_max_*/3287, 2902, 1648, 1617, 1575, 1528, 1499, 1483, 1452, 1432, 1385, 1322, 1272, 1196, 1125, 1043, 936, 885, 834, 817; ^1^H-NMR (DMSO-*d*_6_) δ 8.36 (d, 1H, *J* = 9 Hz), 7.96 (s, 1H), 7.71 (d, 1H, *J* = 9 Hz), 6.87 (d, 1H, *J* = 8 Hz), 6.74 (m, 1H), 6.63 (d, 1H, *J* = 8 Hz), 5.96 (s, 2H), 4.05 (s, 3H); HRMS calcd for C_18_H_14_N_5_O_3_S [M+H]^+^: 380.0817, found 380.0805.

*Methyl 9-(4-bromo-2-fluorophenylamino)thiazolo[5,4-f]quinazoline-2-carbimidate* (**9i**). Purified by flash chromatography eluent (DCM–EtOAc, 2:8) as pale yellow solid (yield: 94%); mp > 260 °C. IR (cm^−1^) ν*_max_* 2950, 1638, 1617, 1595, 1555, 1507, 1479, 1434, 1398, 1352, 1325, 1288, 1224, 1197, 1159, 1115, 1070, 988, 965, 942, 882, 818; ^19^F-NMR (DMSO-*d*_6_) δ −119.8; ^1^H-NMR (DMSO-*d*_6_) δ 8.46 (d, 1H, *J* = 9.0 Hz), 8.02 (s, 1H), 7.69 (d, 1H, *J* = 9.0 Hz), 7.53 (m, 1H), 7.35 (m, 1H), 7.20 (t, 1H, *J* = 9.0 Hz), 3.94 (s, 3H); HRMS calcd for C_17_H_12_N_5_OSBrF [M+H]^+^: 431.9930, found 431.9937.

*Methyl 9-(3-chloro-4-fluorophenylamino)thiazolo[5,4-f]quinazoline-2-carbimidate* (**10i**). Purified by flash chromatography (DCM–EtOAc, 5:5) as orange solid (yield: 98%); mp = 210–212 °C. IR (KBr) ν*_max_*/cm^−1^ 1642, 1559, 1479, 1352, 1260, 1201, 1156, 1073, 942, 817; ^19^F-NMR (DMSO-*d*_6_) δ −126.9; ^1^H-NMR (DMSO-*d*_6_) δ 8.29 (d, 1H, *J* = 9.0 Hz), 8.18 (s, 1H), 7.73 (m, 1H), 7.61 (d, 1H, *J* = 9.0 Hz), 7.30 (m, 1H), 7.27 (t, 1H, *J* = 9.0 Hz), 3.95 (s, 3H); HRMS calcd for C_17_H_12_N_5_OSClF [M+H]^+^: 388.0435, found 388.0447.

### 3.3. In Vitro Kinase Preparation and Assays [19]

#### 3.3.1. Buffers

Buffer A: MgCl_2_ (10 mM), 1 mM ethylene glycol-bis(2-aminoethylether)-*N,N,N',N'*-tetraacetic acid (EGTA), 1 mM dithiothreitol (DTT), 25 mM Tris-HCl pH 7.5, 50 μg heparin/mL.

Buffer B: β-Glycerophosphate (60 mM), 30 mM *p*-nitrophenylphosphate, 25 mM 3-(*N*-morpholino)propanesulfonic acid (Mops) (pH 7.2), 5 mM EGTA, 15 mM MgCl_2_, 1 mM DTT, 0.1 mM sodium vanadate.

#### 3.3.2. Kinase Preparations and Assays

Kinase activities were assayed in triplicates in buffer A or B, for 30 min. at 30 °C, at a final adenosine triphosphate (ATP) concentration of 15 μM. Blank values were subtracted and activities expressed in % of the maximal activity, *i.e.*, in the absence of inhibitors. Controls were performed with appropriate dilutions of dimethylsulfoxide (DMSO). IC_50_ values were calculated from dose-response curves established by Sigma-Plots. The GSK-3, CK1, DYRK1A and CLK1 peptide substrates were obtained from Proteogenix (Oberhausbergen, France).

*CDK5/p25.* (Human, recombinant) was prepared as previously described [20,21]. Its kinase activity was assayed in buffer A, with 1 mg of histone H1/mL, in the presence of 15 μM [γ-^33^P] ATP (3000 Ci/mmol; 10 mCi/mL) in a final volume of 30 μL. After 30 min incubation at 30 °C, 25 μL aliquots of supernatant were spotted onto sheets of Whatman P81 phosphocellulose paper, and 20 s later, the filters were washed eight times (for at least 5 min each time) in a solution of 10 mL phosphoric acid/L of water. The wet filters were counted in the presence of 1 mL ACS (Amersham) scintillation fluid. 

*GSK-3α/β.* (Porcine brain, native) was assayed as described for CDK5/p25, but in buffer A and using a GSK-3 specific substrate (GS-1: YRRAAVPPSPSLSRHSSPHQpSEDEEE) (pS stands for phosphorylated serine) [22].

*CK1δ/ε.* (Porcine brain, native) was assayed as described for CDK5/p25, but using the CK1-specific peptide substrate RRKHAAIGpSAYSITA [23].

*DYRK1A.* (Rat, recombinant, expressed in *E. coli* as a glutathione transferase (GST) fusion protein) was purified by affinity chromatography on glutathione-agarose and assayed, as described for CDK5/p25 using Woodtide (KKISGRLSPIMTEQ) (1.5 µg/assay) as a substrate.

## 4. Conclusions

The convenient synthesis of a forty molecule library of novel 6,6,5-tricyclic thiazolo[5,4-*f*]-quinazolines was realized under microwave irradiation associating Dimroth rearrangement for construction the pyrimidine part and 4,5-dichloro-1,2,3,-dithiazolium chloride (Appel salt) chemistry for introducing the thiazole ring to its quinazoline partner. Our work allowed to prepare in an efficient and reproducible multistep synthesis a novel 6-aminobenzo[*d*]thiazole-2,7-dicarbonitrile (**16**) which can be considered as a very powerful molecular platform for the synthesis of various bioactive derivatives. On chemical and practical aspects this article is a further example illustrating how microwave heating can be a very powerful tool for medicinal chemistry. 

The inhibitory potency of the final products against a panel of four kinases was evaluated. In view of the results of this preliminary study, we consider that the thiazolo[5,4-*f*]quinazoline derivatives (series **7**–**10**) constitute a promising source of inspiration for the synthesis of novel bioactive molecules. Novel synthetic transformations will be explored and factors governing the dual activity of the compounds toward DYRK1A and GSK3 will be further investigated.
